# Law matters – assessment of country-level code implementation and sales of breastmilk substitutes in South Asia

**DOI:** 10.3389/fpubh.2023.1176478

**Published:** 2023-10-23

**Authors:** Constance Ching, Vani Sethi, Tuan Thanh Nguyen, Zivai Murira, Katherine Shats, Dhammica Rowel, Khadheeja Ahmed, Kinley Dorji, Indrani Chakma, Karan Courtney Haag, Phulgendra Prasad Singh, Salma Khatoon, Uzma Khurram Bukhari, Ahmadwali Aminee, Sebanti Ghosh, Thomas Forissier, Kristen Kappos, Paul Zambrano, Golam Mohiuddin Khan

**Affiliations:** ^1^Alive & Thrive, Global Nutrition, FHI 360, Hanoi, Vietnam; ^2^UNICEF Regional Office for South Asia, Kathmandu, Nepal; ^3^UNICEF Headquarters, New York, NY, United States; ^4^UNICEF Sri Lanka Country Office, Colombo, Sri Lanka; ^5^UNICEF Maldives Country Office, Malé, Maldives; ^6^UNICEF Bhutan Country Office, Thimphu, Bhutan; ^7^UNICEF Nepal Country Office, Kathmandu, Nepal; ^8^UNICEF Field Office for Khyber-Pakhtunkhwa, Peshawar, Pakistan; ^9^UNICEF Field Office for Punjab Field Office, Lahore, Pakistan; ^10^UNICEF Office for Afghanistan, Kabul, Afghanistan; ^11^Alive & Thrive, Global Nutrition, FHI360, New Delhi, India; ^12^Alive & Thrive, Global Nutrition, FHI 360, Washington, DC, United States; ^13^Alive & Thrive, Global Nutrition, FHI 360, Manila, Philippines; ^14^UNICEF Bangladesh Office, Dhaka, Bangladesh

**Keywords:** baby foods, baby milks, breastmilk substitutes, commercial milk formula, inappropriate marketing, legal measures, South Asia, breastfeeding

## Abstract

**Objectives:**

This study examines the status of implementation of the International Code of Marketing of Breast-milk Substitutes of eight countries in the South Asia region (Afghanistan, Bangladesh, Bhutan, India, Maldives, Nepal, Pakistan, and Sri Lanka), and describes the sales value and volume of commercial milk formula (CMF) marketed as breastmilk substitutes (BMS) and baby food in four countries (Bangladesh, India, Pakistan, and Sri Lanka).

**Design:**

A mix of descriptive methods is used to assess national status of Code implementation, including a desk review of the 2022 WHO/UNICEF/IBFAN Code Status Report, systematic content analysis of national Code measures, and insights generated from the participation of key government and UNICEF/WHO actors in a regional workshop that aimed to identify each country’s barriers, gaps, and the status of Code implementation. Data on the sales value and volume of CMF and baby food between 2007 to 2021 and with the prediction to 2026 in Bangladesh, India, Pakistan, and Sri Lanka were obtained from Global Data.

**Findings:**

There are major gaps in Code implementation in countries even with legal measures considered substantially aligned with the Code, such as the inadequate age range of CMF covered in the scope, insufficient safeguards against conflicts of interest in the health system, lack of warning of risks of intrinsic contamination of powdered milk formula, and an absence of effective monitoring and enforcement mechanisms. Data on CMF sales shows health facilities and pharmacies sustain the highest sales. Lower sales volume of infant formula (including special formula), compared to other CMF such as follow-up formula and growing-up milk, has been observed in three of the four countries (Bangladesh, India, and Sri Lanka). Overall, GUM, followed by baby cereals, accounted for a large portion of CMF and baby foods sales in the same three countries.

**Recommended actions include:**

(1) Closing the gaps between national measures and the Code, (2) Ensuring effective monitoring and enforcement mechanisms, (3) Strengthening conflicts of interest safeguards in the health system, (4) Tackling digital marketing, and (5) Galvanizing political support and support from in-country public health and women’s rights jurist networks.

## 1. Introduction

Close to 900,000 newborns die in South Asia each year. The region accounts for almost 40% of the total global neonatal deaths (1). Breastfeeding is one of the most effective ways to ensure child survival and development – with health protection persisting until later in life (2). However, the unethical and predatory marketing of commercial milk formula (CMF) as breastmilk substitutes (BMS),^1^ complementary foods, bottles and teats is a main barrier toward improving breastfeeding (3).

Inadequate breastfeeding is associated with increased risks of infectious and non-communicable illnesses, food insecurity, economic loss, morbidity, and mortality (2, 4–6). The two-fold CMF global sales increase in the past two decades, which currently reaches US$55.6 billion per year, suggests the industry’s marketing strategies have been effective in displacing breastfeeding (7, 8). In South Asia, less than 40% infants are breastfed within the first hour of birth, only 61% are exclusively breastfed during the first 6 months (9). Given the region’s child mortality rates and food insecurity risks, efforts to improve breastfeeding in the South Asia region are critical (1, 10–12).

The World Health Assembly (WHA) adopted the International Code of Marketing of Breast-milk Substitutes in 1981 (the 1981 Code) out of a global concern that aggressive and inappropriate marketing of CMF as BMS and bottles and teats were contributing to a sharp decline on breastfeeding and an increase in child morbidity and mortality (13). Together with subsequent relevant WHA resolutions (collectively known as “the Code” hereinafter), it aims to prohibit promotion that undermines breastfeeding and appropriate infant and young child feeding (IYCF). It also serves to protect bottle-fed infants and children by ensuring information on health hazards, appropriate use, and safe preparation (14). The products covered by the Code are products marketed for the feeding of infants and children up to the age of 36 months (including CMF and foods for infants and young children) and feeding bottles and teats (see Tables 1, 2 for details). Although CMF for pregnant and lactating women does not come under the scope of the Code, it has become very popular and is used as a gateway to promote CMF for infants and young children (15). Governments are obligated to adopt the Code as minimum standards in the form of legal measures (13). Seven out of eight countries in South Asia have, to varying degrees, adopted the Code into national legal measures. Like the rest of the world (3, 8, 16, 17), the region’s ongoing inappropriate marketing that violates the Code and national laws has been evident and rampant (Table 3) (18–23). There is growing evidence of highly insidious practices such as companies co-opting health professionals and the health systems through sponsorships and other financial links, and industry’s ongoing lobbying with governments to weaken national regulatory measures (24, 25).

**Table 1:** Summary of the International Code of Marketing of Breast-milk Substitutes.

Aim	To contribute to the provision of safe and adequate nutrition for infants by the protection and promotion of breastfeeding and the proper use of breastmilk substitutes, when these are necessary, on the basis of adequate information and through appropriate marketing and distribution
Scope	Applies to breastmilk substitutes* ^1^ or any food being marketed or otherwise represented as a partial or total replacement for breastmilk. This includes: Infant formula. Follow-up formula (sometimes referred to as ‘follow-on milk1).* Growing-up milk.* Any other milk for children 0 < 36 months.* Any other food or liquid (such as cereal, jarred food, infant tea, juice and mineral water) that is represented as suitable to be fed to infants less than 6 months of age* The International Code also applies to feeding bottles and teats. Restrictions for foods for infants and young children (6–36 months) are addressed in the WHO Guidance on ending the inappropriate promotion of foods for infants and young children.
Promotion	No advertising or promotion of above products to the public. No nutrition or health claims on products.*^ ^2^
Samples	No free samples to mothers, their families, or health care workers.
Health care facilities	No promotion of products, i.e., no product displays, posters, calendars, or distribution of promotional materials. No mothercraft nurses or similar corporation-paid personnel.
Health care workers	No gifts or samples to health care workers. Financial support and incentives should not create conflicts of interest. ^ ^3^ Feeding with infant formula, whether manufactured or home-prepared, should be demonstrated only by health workers, or other community workers if necessary.
Supplies	No free or low-cost supplies of breastmilk substitutes to any part of the health care system.^ ^4^
Information	Information and education materials must explain the benefits of breastfeeding, the health hazards associated with bottle-feeding, and the costs of using infant formula. Product information must be factual and scientific. Governments to avoid conflicts of interest so materials under infant and young child programs should not be sponsored by manufacturers and distributors.^ ^5^
Labels	Product labels must clearly state the superiority of breastfeeding, the product should be used only on the advice of a health worker as to the need for its use and the proper method of use, and a warning about health hazards. No pictures of infants, other pictures, or text idealizing the use of infant formula. Labels must contain the warning that powdered infant formula may contain pathogenic microorganisms and must be prepared and used appropriately. ^ ^5^ Labels on complementary foods should not cross-promote breastmilk substitutes, should not promote bottle-feeding, and should state the importance of continued breastfeeding.^ ^6^
Quality	Unsuitable products, such as sweetened condensed milk, should not be promoted for babies. All products should be of a high quality (Codex Alimentarius Standards) and take account of the climatic and storage conditions of the country where they are used.
Monitoring and implementation	Governments, with support from WHO/UNICEF, are responsible for monitoring the compliance of the Code. NGOs, professional groups, institutions, consumer organizations, and individuals should inform governments and companies about violations of Code. Independently of any other measures taken for implementation of the Code, companies are responsible for monitoring their marketing practices and to ensure that their conduct at every level conforms to it. Governments are obligated to implement the Code through legal measures as a minimum standard.

(*) denotes products and definitions which are clarified by the WHO Guidance on ending the inappropriate promotion of foods for infants and young children Guidance A69/7 Add.1 which was welcomed by WHA Resolution 69.9 [2016].

(^) denotes that Code provisions have been clarified and extended by subsequent World Health Assembly Resolutions which are summarized in Annex B.

^1^ WHA49.15 [1996], WHA54.2 [2001] and WHA63.23 [2010].

^2^ WHA58.32 [2005] and WHA63.23 [2010].

^3^ WHA49.15 [1996] and WHA58.32 [2005].

^4^ WHA47.5 [1994] and WHA58.32 [2005].

^5^ WHA58.32 [2005].

^6^ A69/7 Add.1.

**Table 2:** Relevant WHA resolutions: key points.

Year	Resolution	Key points
1981	WHA 34.22	Stresses that adoption and adherence to the Code is a minimum requirement. Member States are urged to implement the Code into national legislation, regulations and other suitable measures.
1982	WHA35.26	Recognizes that commercial promotion of breastmilk substitutes contributes to an increase in artificial feeding and calls for renewed attention to implement and monitor the Code at national and international levels.
1984	WHA37.30	Requests that the Director General work with Member States to implement and monitor the Code and to examine the promotion and use of foods unsuitable for infant and young child feeding.
1986	WHA39.28	Urges Member States to ensure that the small amounts of breastmilk substitutes needed for a minority of infants are made available through normal procurement channels and not through free or subsidized supplies. Directs attention of Member States to the following: 1. Any food or drink given before complementary feeding is nutritionally required may interfere with breastfeeding and therefore should neither be promoted nor encouraged for use by infants during this period; 2. The practice of providing infants with follow up milks is “not necessary.”
1988	WHA41.11	Requests the Director General to provide legal and technical assistance to Member States in drafting or implementing the Code into national measures
1990	WHA43.3	Highlights the WHO/UNICEF statement on “protection, promoting and supporting breastfeeding: the special role of maternity services” which led to the Baby-Friendly Hospital Initiative in 1992. Urges Member States to ensure that the principles and aim of the Code are given full expression in national health and nutrition policy and action.
1994	WHA47.5	Reiterates earlier calls in 1986, 1990 and 1992 to end “free or low-cost supplies” and extends the ban to all parts of the health care system. Provides guidelines on donation of breastmilk substitutes in emergencies.
1996	WHA49.15	Calls on Member States to ensure that: 1. complementary foods are not marketed for or used to undermine exclusive and sustained breastfeeding; 2. financial support to health professionals does not create conflicts of interests; 3. Code monitoring is carried out in an independent, transparent manner free from commercial interest.
2001	WHA 54.2	Sets global recommendation of “6 months” exclusive breastfeeding, with safe and appropriate complementary foods and continued breastfeeding for up to two years or beyond.
2002	WHA55.25	Endorses the Global Strategy on Infant and Young Child Feeding which confines the baby food manufacturers and distributors’ role to: 1. ensuring quality of their products; 2. complying with the Code and subsequent WHA resolutions, as well as national measures. Recognizes the role of optimal infant feeding to reduce the risk of obesity. • Alerts that micronutrient interventions should not undermine exclusive breastfeeding.
2005	WHA58.32	Asks Member States to: 1. ensure that nutrition and health claims for breastmilk substitutes are not permitted unless national/regional legislation allows; 2. be aware of the risks of intrinsic contamination of powdered infant formulas and to ensure this information be conveyed through label warnings; 3. ensure that financial support and other incentives for programs and health professionals working in infant and young child health do not create conflicts of interest.
2006	WHA59.11	Member States to make sure the response to the HIV pandemic does not include non-Code compliant donations of breastmilk substitutes or the promotion thereof.
2006	WHA59.21	Commemorates the 25th anniversary of the adoption of the Code; welcomes the 2005 Innocenti Declaration and asks WHO to mobilize technical support for Code implementation and monitoring.
2008	WHA61.20	Urges Member States to: 1. scale up efforts to monitor and enforce national measures and to avoid conflicts of interest; 2. investigate the safe use of donor milk through human milk banks for vulnerable infants, mindful of national laws, cultural and religious beliefs.
2010	WHA63.23	Urges Member States to: 1. strengthen implementation of the Code and resolutions, the Global Strategy on Infant and Young Child Feeding, the Baby-Friendly Hospital Initiative, the Operational Guidance for Emergency Relief Staff; 2. end all forms of inappropriate promotion of foods for infants and young children and that nutrition and health claims should not be permitted on these foods. Urges corporations to comply fully with responsibilities under the Code and resolutions.
2012	WHA65.6	Urges Member States to put into practice the comprehensive implementation plan on maternal, infant and young child nutrition, including: 1. developing or strengthening legislative, regulatory or other measures to control the marketing of breastmilk substitutes; 2. establishing adequate mechanisms to safeguard against potential conflicts of interest in nutrition action. Requests the Director General to: 1. provide clarification and guidance on the inappropriate promotion of foods for infants and young children as mentioned in WHA63.23; 2. develop processes and tools to safeguard against possible conflicts of interest in policy development and implementation of nutrition programs.
2014	WHA67(9)	Infant and Young Child Nutrition (MIYCN) Plan which includes increasing the rate of exclusive breastfeeding to at least 50% by 2025 as a global target. The indicator for regulation of marketing is the number of countries with legislation or regulations fully implementing the Code and Resolutions.
2016	WHA69.9	This Resolution welcomes the WHO Guidance on ending the inappropriate promotion of foods for infants and young children. It calls upon 1. Member States to take all necessary measures to implement the Guidance 2. Manufacturers and distributors of foods for infants and young children to adhere to the Guidance. The Guidance clarified that follow-up milks and growing up milks are covered by the Code and should be treated as such when implementing the Code. The Guidance also recommends that there should be no cross-promotion to promote breastmilk substitutes via the promotion of foods for infants and young children. Practices that constitute conflicts of interest in the health system were also discussed at length and their prohibition recommended.
2018	WHA71.9	This Resolution urges Member States to: 1. reinvigorate the Baby-friendly Hospital Initiative and the full integration of the revised 10 Steps to Successful Breastfeeding which incorporates Code compliance in Step 1; 2. take all necessary measures to implement recommendations to end the inappropriate promotion of foods for infants and young children.
2020	WHA73.26	Requests the Director-General to review current evidence and prepare a comprehensive report on the scope and impact of digital marketing strategies for the promotion of breast-milk substitutes to the 75th World Health Assembly in 2022.
2022	WHA75.21	Requests the Director-General to develop guidance for Member States on regulatory measures to restrict the digital marketing of breastmilk substitutes, to ensure existing and new regulations designed to implement the International Code of Marketing Breast-milk Substitutes and relevant WHA resolutions adequately address digital marketing practices, and report progress in the 77th WHA 2024.

**Table 3:** Inappropriate marketing practices/code violations documented in studies in South Asia.

Marketing practices documented in studies	Country	Study
Free BMS samples were distributed to health workers and mothers. Gifts were provided to health workers within health facilities in Bangladesh.	Bangladesh	Taylor (18)
Companies offered gifts, free samples and sponsorship to health workers, most of whom were not aware of the national regulations that give effect to the Code.	Pakistan	Salasibew(19)
Labeling violations found (e.g., health claims and idealization).	India, Maldives, Nepal, and Sri Lanka	IBFAN(20)
Rampant promotion on online shopping portals	India	
Labeling and points of sale (retail outlets) violations were prevalent.	Bangladesh	Sheikh et al. (21)
Companies exploited the COVID-19 pandemic to use social media to contact mothers.	India	Ching, et al. (22)
Donated breastmilk substitutes to the Provincial Disaster Management Authority (during the COVID-19 pandemic). Digital marketing of breastmilk substitutes in Pakistan made refence to COVID-19, claiming their BMS products can help boost immunity.	Pakistan	
BMS companies committed inappropriate marketing in different sectors, such as promotion to the public, inappropriate labeling, and engagement with health workers.	India	BPNI (21)

Apart from a study that contrasted CMF sales to information on Code implementation and breastfeeding practices between India and China (26), there is a research gap regarding studies that assess the relationship between implementation of the Code and the sales of CMF and foods for infants and young children. Thus, this study seeks to examine the extent and nature of national implementation of the Code in eight countries in the South Asia region (Afghanistan, Bhutan, Bangladesh, India, Maldives, Nepal, Pakistan and Sri Lanka) and trends in CMF and baby food sales in four countries with available sales data (Bangladesh, India, Pakistan, and Sri Lanka).

## 2. Methods

### 2.1. Status of code implementation: review of legal measures

This study was conducted between June–December 2022,^2^ on countries under the geographical scope of the UNICEF Regional Office for South Asia (UNICEF ROSA): Afghanistan, Bhutan, Bangladesh, India, Maldives, Nepal, Pakistan and Sri Lanka. It uses a mix of descriptive analytical methods:

- Desk review of Code implementation status of each country in the 2022 WHO/UNICEF/IBFAN Marketing of breast-milk substitutes: National implementation of the International Code Status Report (the 2022 Code Status Report) (17).
- Systematic content analyses (27) of content of all relevant provisions in the national Code measures, including gaps in comparison with provisions in the Code.

#### 2.1.1. Data collection

Data was extracted from two major sources:

1. The 2022 WHO/UNICEF/IBFAN Marketing of breast-milk substitutes: National implementation of the International Code Status Report (the 2022 Code Status Report) (17): It presents the legal status and the extent to which the provisions of the Code, WHA resolutions, and the recommendations contained in the 2016 WHO Guidance have been incorporated in national legal measures. The national measures were analyzed for scope and content using the report’s own standardized checklist divided into seven sections: scope; monitoring and enforcement; informational/ educational materials on infant and young child feeding (IYCF); promotion to the general public; promotion in health facilities; engagement with health workers and systems; and labeling.
  A scoring algorithm has been used to classify national measures into four categories, with a maximum possible total of 100 (See Tables 4, 5).

- Substantially aligned with “the Code”: countries have enacted legislation or adopted regulations, decrees or other legally binding measures encompassing a significant set of provisions of the Code (score of 75–100).
- Moderately aligned with “the Code”: countries have enacted legislation or adopted regulations, decrees or other legally binding measures encompassing a majority of provisions of the Code (score of 50 - < 75).
- Some provisions of “the Code” included: countries have enacted legislation or adopted regulations, decrees or other legally binding measures covering less than half of the provisions of the Code (score of <50).
- No legal measures: countries have taken no action or have implemented the Code only through voluntary agreements or other non-legal measures (includes countries that have drafted legislation but not enacted it).

**Table 4:** Overall Code implementation status, subtotal, and total scores^1^.

Category	Afghanistan	Bangladesh	Bhutan	India	Maldives	Nepal	Pakistan	Sri Lanka
Overall implementation (alignment with the Code)	Substantial	Substantial	No measures	Substantial	Substantial	Moderate	Moderate	Moderate
Overall implementation total score (/100)	92	79	-	79	94	71	73	69
Scope (/20)	18	20	-	16	20	16	16	16
Monitoring and enforcement (/ 10)	10	8	-	8	10	8	5	8
Informational/educational materials (/ 10)	10	6	-	4	6	6	6	2
Promotion to public (/ 20)	17	20	-	20	20	17	20	20
Promotion in health care facilities (/ 10)	10	10	-	10	10	10	10	10
Engagement with health workers and systems (/ 15)	14	4	-	13	14	7	10	8
Labeling (/ 15)	14	11	-	8	14	7	6	5
Year of adoption	2009	1984	-	1992	2008	1992	2002	1983
Year of latest review/amendment	2009	2017	-	2003	2008	1994	2018	2003

^1^Adapted from the WHO/UNICEF/IBFAN Code implementation report 2022.

Substantial, substantially aligned with the Code.

Moderate, moderately aligned with the Code.

No measures, no legal measures.

**Table 5:** Implementation status by Code and WHA resolutions provision^1^.

Category	Provisions covered	Afghanistan	Bangladesh	India	Maldives	Nepal	Pakistan	Sri Lanka
Scope	BMS products covered up to age (months)	Unspecified	60	24	36	12	12	12
	Complementary foods covered	**√**	**√**	**√**	**√**	**√**	**√**	**√**
	Bottles and teats covered	**√**	**√**	**√**	**√**	**√**	**√**	**√**
Monitoring and enforcement	Identifies who is responsible for monitoring compliance	**√**	**√**	**√**	**√**	**√**	**×**	**√**
	Defines sanctions for violations	**√**	**√**	**√**	**√**	**√**	**√**	**√**
	Requires that monitoring and enforcement should be independent, transparent and free from commercial influence	**√**	**×**	**×**	**√**	**×**	**×**	**×**
Provisions on informational/ educational/ communication materials	Required content for all information/education/communication materials:							
	Informational/educational materials from industry prohibited	**√**	**√**	**×**	**×**	**×**	**√**	**√**
	the benefits and superiority of breastfeeding	**√**	**√**	**√**	**√**	**√**	**×**	**×**
	maternal nutrition and preparation for and maintenance of breastfeeding	**√**	**√**	**√**	**√**	**√**	**×**	**×**
	the negative effect on breastfeeding of introducing partial bottle-feeding	**√**	**√**	**√**	**√**	**√**	**×**	**×**
	the difficulty of reversing the decision not to breastfeed	**√**	**√**	**√**	**√**	**√**	**×**	**×**
	proper use of infant formula	**√**	**×**	**×**	**√**	**√**	**×**	**×**
	Required content for materials dealing with infant formula:							
	social and financial implications of its use	**√**	**√**	**√**	**√**	**√**	**×**	**×**
	health hazards of inappropriate feeding	**√**	**√**	**√**	**√**	**√**	**×**	**×**
	health hazards of inappropriate use	**√**	**√**	**√**	**√**	**√**	**×**	**×**
	risk of intrinsic contamination of powdered formula	**×**	**×**	**×**	**×**	**×**	**×**	**×**
	Prohibited content							
	1. Prohibition of reference to proprietary products	**√**	**×**	**√**	**√**	**√**	**×**	**×**
	2. Prohibition of pictures or text idealizing BMS	**√**	**×**	**×**	**√**	**√**	**√**	**√**
Provisions on promotion to general public	Advertising	**√**	**√**	**√**	**√**	**√**	**√**	**√**
	Samples to public	**√**	**√**	**√**	**√**	**√**	**√**	**√**
	Promotional devices at point of sale	**√**	**√**	**√**	**√**	**√**	**√**	**√**
	Gifts to pregnant women and mothers	**√**	**√**	**√**	**√**	**√**	**√**	**√**
	Contact with mothers	**×**	**√**	**√**	**√**	**√**	**√**	**√**
Provisions on promotion in health care facilities	Overall prohibition on use of health care facilities for promotion	**√**	**√**	**√**	**√**	**√**	**√**	**√**
	Types of prohibition explicitly covered							
	Display of covered products	**√**	**×**	**×**	**√**	**√**	**√**	**√**
	display of placards or posters concerning covered products	**√**	**√**	**√**	**√**	**√**	**√**	**√**
	distribution of any material provided by a manufacturer or distributor	**√**	**×**	**√**	**×**	**√**	**×**	**√**
	use of health facility to host events, contests or campaigns	**√**	**√**	**×**	**×**	**×**	**×**	**×**
	use of personnel provided by or paid for by manufacturers and distributors	**×**	**√**	**√**	**√**	**×**	**×**	**√**
Engagement with health workers and systems	Overall prohibition of all gifts or incentives to health workers and health systems	**√**	**×**	**√**	**√**	**√**	**√**	**×**
	Type of gift or incentive:							
	financial or material inducements to promote products within the scope	**√**	**√**	**√**	**√**	**√**	**√**	**√**
	fellowships, study tours, research grants, attendance at professional conferences	**√**	**√**	**√**	**√**	**×**	**√**	**√**
	Fellowships, etc., not permitted must be disclosed to the institution	**×**	**×**	**×**	**×**	**√**	**×**	**×**
	Other prohibitions							
	Provision of free or low-cost supplies in any part of the health care system	**√**	**×**	**√**	**√**	**×**	**√**	**√**
	Donations of equipment or services	**×**	**×**	**×**	**×**	**×**	**×**	**×**
	Donations prohibited only if they refer to a proprietary product	**√**	**×**	**×**	**√**	**×**	**√**	**×**
	Product samples	**√**	**√**	**√**	**√**	**√**	**√**	**√**
	Product information restricted to scientific and factual matters	**√**	**×**	**√**	**√**	**√**	**√**	**√**
	Sponsorship of meetings of health professionals or scientific meetings	**√**	**×**	**√**	**√**	**×**	**×**	**×**
Provisions on labeling	Prohibition of nutrition and health claims	**√**	**√**	**×**	**√**	**×**	**×**	**×**
	Required information on infant formula products							
	The words “Important Notice”	**√**	**√**	**√**	**√**	**√**	**×**	**√**
	Statement on superiority of breastfeeding	**√**	**√**	**√**	**√**	**√**	**√**	**√**
	Statement on use only on the advice of a health worker	**√**	**×**	**√**	**×**	**√**	**×**	**√**
	Instructions for appropriate preparation	**√**	**√**	**√**	**√**	**√**	**√**	**√**
	Warning against the health hazards of inappropriate preparation	**√**	**√**	**√**	**√**	**×**	**×**	**×**
	Warning that powdered formula may contain pathogens	**×**	**√**	**×**	**√**	**×**	**×**	**×**
	Prohibited content for infant formula							
	Pictures that may idealize the use of infant formula	**√**	**√**	**√**	**√**	**√**	**√**	**√**
	Required information for follow-up formula, growing up milks, as well as other foods for IYC up to 3 years							
	Recommended age for introduction of the product	**√**	**×**	**×**	**√**	**×**	**×**	**×**
	Importance of continued breastfeeding for 2+ years	**√**	**×**	**×**	**√**	**×**	**×**	**×**
	Importance of no complementary feeding before 6 months	**√**	**×**	**×**	**√**	**×**	**×**	**×**
	Prohibited content for follow-up formula, growing up milks, as well as other foods for IYC up to 3 years							
	Images/texts suggesting use before 6 months	**√**	**√**	**×**	**√**	**√**	**√**	**×**
	Images/text that undermines/discourages breastfeeding or compares to breast milk	**√**	**×**	**×**	**√**	**√**	**√**	**×**
	Messages that recommend or promote bottle feeding	**×**	**×**	**√**	**×**	**×**	**×**	**×**
	Professional endorsements	**√**	**√**	**√**	**√**	**×**	**×**	**×**

^1^Adapted from the WHO/UNICEF/IBFAN Code implementation report 2022.

*Bhutan is not included in this table as there are no legal measures for assessment.

Subsequent Annexes in the 2022 Code Status report (Annexes 2–9) provide a further detailed analysis of Legal status of the Code, total and category sub-total scores, provisions on scope and on monitoring and enforcement, provisions on informational and educational materials, provisions on promotion to the general public, provisions on promotion in health facilities, provisions on engagement with health care workers and health systems and provisions on labeling. The relevant Code legal measures of Afghanistan, Bangladesh, India, Maldives, Nepal, Pakistan, and Sri Lanka; and the 2002 National Policy Statement of Bhutan.^3^ First, a list of all relevant legal measures was extracted from the 2022 Code Status Report (17). The list was verified by participants representing the eight countries in a Regional Workshop for a Bottleneck Analysis of Implementation of Legal Measures, Monitoring and Enforcement Systems to Protect Breastfeeding in South Asia that was held in Colombo, Sri Lanka, from 14 to 16 November 2022 (28). After verification, the legal measures of the seven countries and National Policy Statement of Bhutan were obtained from the UNICEF ROSA and relevant country offices.

#### 2.1.2. Coding and analysing the data

A systematic content analysis (SCA) (27) was used to review provisions in the national Code measures and compare those with the provisions in the Code.

*A priori* coding was conducted using the categories outlined in the 2022 Code Status Report’s scoring algorithm (Table 4): scope, monitoring and enforcement, informational/ educational/ communication materials, provisions on promotion in health facilities, engagement with health workers and systems, and provisions on labeling. Coders (first and second authors) applied the process outlined by Salehijam (27): First-level coding involved examining the text of all relevant national measures identified and collected, and then separating the data (text) into the different categories based on their content. Additional steps were taken to ensure the first-level codes were non-duplicative and contained sub-categories for further fine-tuning. The second-level coding was to combine, where appropriate, some first-level codes into final codes, for instance, “Promotion in health facilities” and “Engagement with health workers and systems” were combined as “Health facilities and health workers.” Third-level coding involved applying the final codes to the relevant text (provisions) in the International Code and all relevant WHA resolutions, and then summarizing the data into key points. A constant comparison technique, which inductively and continuously compares emerging categories with previous ones until saturation, was used to compare data in each category of the national legal measures with those in the Code and relevant WHA resolutions, to identify alignments, gaps, and areas where national legal measures provide stronger protection against promotion of CMF and related products compared to the Code. To strengthen inter-coder objectivity and reliability, the coded data was reviewed by the third author at each level of coding. Discrepancies were reviewed until consensus was reached among the research team to establish a single application of final codes.

#### 2.1.3. Additional information

Information emerged from the Regional Workshop for a Bottleneck Analysis of Implementation of Legal Measures, Monitoring and Enforcement Systems to Protect Breastfeeding in South Asia^4^ (referred to as the “2022 Regional Workshop” hereafter) held in Sri Lanka in November 2022, was included to triangulate and contextualize the findings. The information includes:

1. Input from the participants, in particular the status of monitoring and enforcement in a country (28).
2. The Regional Legal Desk Review: a review of national measures of the eight countries as part of the reference materials for the regional workshop (30).
3. The SWOT Analysis conducted by country participants during the Regional Workshop with the aim to identify inherent strengths and weaknesses, opportunities for improvement, and threats present in the internal and external environments that could potentially threaten effective Code implementation^5^ (28).

### 2.2. Baby food and CMF sales

Data on the sales value and volume of baby food and CMF marketed as BMS between 2007 and 2021 and with the prediction to 2026 were obtained from Global Data^6^ (31), a data analytics company that offers bespoke market reports and forecasts through a license of UNICEF Regional Office for South Asia (ROSA). Related data was extracted from GlobalData using its pivot function to generate Microsoft Excel spreadsheets, with market value and volume, segments of CMF, per-capita consumption and expenditure, and distribution channels of baby food and CMF as key indicators.

The information on Asia-Pacific and the world is only available in 2015 and 2026, hence some findings used only three data points because the relative estimates were used to compare between the region and the world. As provided by Global Data, all values have been standardized using the 2015 data to allow comparison across the years. Data were only available in four South Asian countries: Bangladesh, India, Pakistan, and Sri Lanka.

The baby food category includes baby drinks, CMF, baby wet meals, baby finger food, baby cereals, and dry meals. The CMF category is further stratified to include special formula and starter/first stage formula (which have the same age designation and are commonly known as infant formula/IF), follow-on milk /second stage (which have the same age designation and are commonly known as follow-up formula/FUF), and toddler milk/third stage (which have the same age designation and are commonly known as growing-up milk/GUM).^7^

Microsoft Excel Pivot tables and Figures were used to extract relevant findings from the Excel spreadsheet to generate appropriate tables and figures for this manuscript. Since this is a descriptive analysis, we did not perform any statistical testing. Nonetheless, we visually examined the potential relationship between CMF sales with the implementation of national Code-based measures. The trends of the studied indicator and proposed potential ecological association were visually examined, with the aim to understand the relationship between an outcome and an exposure at a population level. However, the ecological study design provided weaker causal inference compared to other epidemiological studies (32).

## 3. Results

### 3.1. Status of code implementation

This section includes the analysis of national measures that give effect to the Code in the eight countries.

#### 3.1.1. Afghanistan

##### 3.1.1.1. Background and scope

Afghanistan adopted the Regulation on Support and Promotion of Breastfeeding in 2009 with clear objectives to protect the health and safety of the child and mother, encourage and protect breastfeeding and appropriate complementary feeding, and ensure the proper use of infant feeding (and other related) products. The Regulation does not have a specified age for the coverage of CMF and complementary food products but defines “baby” as a new-born up to 30 months, departing from the internationally agreed age range of 0–12 months for infants. Many of the provisions address marketing products for “babies.” The Code covers CMF products up to 36 months, and the 30-month scope of the Regulation may provide loopholes for CMF (e.g., GUMs) and foods for infants and young children to be promoted from 30 months on. The Regulation covers pacifiers, which are not explicitly covered in the Code. Afghanistan’s regulation is considered as being substantially aligned with the Code, scoring 92 out of 100 points in total.

##### 3.1.1.2. Informational and educational materials

The Regulation is most aligned with the Code on provisions regarding informational and educational materials and labeling compared to all countries in the region. It includes provisions addressing prohibition of company produced or sponsored materials, and all required statements for CMF in general and IF, such as benefits of breastfeeding, maternal nutrition and preparation, negative effects of introducing bottle-feeding, and health hazards of inappropriate feeding. It also includes provisions that prohibit reference made to proprietary products and idealizing pictures and text. Recommended age and prohibitions of marketing complementary foods and FUF and GUM to infants less than 6 months are included.

One of the major gaps of the Regulation is the absence of provision to ensure a warning on the risk of intrinsic contamination of powdered IF in communications materials.

##### 3.1.1.3. Promotion to general public

The Regulation has fairly comprehensive safeguards restricting promotion to general public, (e.g., advertising, samples, promotion at retail outlets), however, there is no explicit ban on company contact with mothers.

##### 3.1.1.4. Health facilities and health workers

There are general restrictions on using health facilities for promotion, including the ban on distribution of company materials, free or low-cost supplies. The Regulation also includes provisions that ban the use of health facilities to host company events, phone counseling services, contests, or campaigns. It however does not explicitly ban the use of personnel associated with companies such as company sponsored mothercraft nurses. Even though the Regulation offers considerable protection from conflicts of interest in the health system, it does not prohibit all donations of equipment by companies, permitting such donations as long as they do not refer to a proprietary product. Although there are overall restrictions on gifts and financial inducements to health workers to promote products covered in the national law, the restrictions on fellowships and grants are not a ‘blanket’ ban, and only “private gifts, aids or other kind of benefits” that are associated with company sponsorship are banned.

##### 3.1.1.5. Labeling

The Regulation has strong labeling provisions, prohibiting all graphics and images except for those used to illustrate methods of preparation. It also prohibits nutrition or health claims, although this prohibition only applies to labels of IF and FUF, leaving it open to manufacturers to make such claims in relation to GUMs or complementary foods.

Endorsement by professional associations, as well as idealizing claims on standards or quality are not allowed. The Regulation is aligned with the Code in all prohibited and required content, except for a warning statement on the risks of intrinsic contamination. Cup-feeding is recommended as the preferred method of artificial feeding. For FUF and complementary food, the label must state it is inappropriate to use before 6 months, and for complementary food, there must be a statement on importance of continued breastfeeding up to 2 years and inappropriate complementary feeding that includes nutrient-rich family foods. The Regulation also stipulates that labels of pacifiers are to include a warning that its use interferes with breastfeeding, and for labels of feeding bottles and teats to carry a warning that children should not be left alone to self-feed for long periods of time.

##### 3.1.1.6. Monitoring and enforcement

There are mechanisms to ensure monitoring takes place independently. The Regulation provides for the establishment of a “National Committee for support and promotion of breastfeeding” and that the Committee “shall appoint Professionals as monitors to monitor better implementation of the regulation.” It is unclear whether such monitors have been appointed, leaving companies relatively free to violate the national regulations with impunity.

#### 3.1.2. Bangladesh

##### 3.1.2.1. Background and scope

Bangladesh adopted its Breastmilk Substitutes (Regulation of marketing) Ordinance in 1984, one of the first countries in the region to adopt legal measures to implement the Code. However, there were some major gaps between the Ordinance and the Code. Subsequently, the 2013 Breast-milk Substitutes, Infant Foods, Commercially Manufactured Complementary Foods and the Accessories Thereto (Regulation of Marketing) Act (the Act) was adopted to introduce several new provisions, bringing it closer to the Code. The 2017 Breast-milk Substitutes, Infant Foods, Commercially Manufactured Complementary Foods and the Accessories Thereof (Regulation of Marketing) Rules (the Rules) strengthened the provisions on educational and informational materials. The Act and the Rules combined have included many relevant Code provisions, and although it is substantially aligned with the Code, there are weaknesses which make implementation and enforcement challenging. Implementation of the Act falls under the jurisdiction of the Director of Institute of Public Health Nutrition, receiving advice from a nine-member National Advisory Committee. In terms of legal status of the Code, Bangladesh is considered as being “Substantially aligned with the Code,” scoring 79 out of a possible 100 points.

The 2022 Code Status Report indicates that the Bangladesh regulations cover CMF up to the age of 60 months, but this is not clear from the text of the 2017 Rules. The scope defines “breastmilk substitutes” as “any infant food for a child up to 6 months,” whereas the Code defines BMS as CMF products marketed as suitable for a child up to 36 months. The Act defines “infant foods” as “any food for a child above the age of 6 months” marketed as a partial or total replacement of breastmilk, without an upper limit. The lack of clarity in definition, especially the definition for CMF which has a six-month upper age limit, can create confusion that panders to the narrow interpretation favored by industry. Meanwhile, the Act restricts promotion of “commercially manufactured complementary foods” (foods for infants and young children) up to 5 years, which goes beyond the 2016 Guidance’s scope of 3 years. Even though the Rules were adopted in 2017 to strengthen and clarify the Act, the definitions of products in the scope remain unchanged.

##### 3.1.2.2. Informational and educational materials

The 2017 Rules have strengthened the provisions on educational and informational materials. The Act and the Rules combined have included many relevant Code provisions. The relevant provisions are focused on ensuring information on the benefits and optimal duration of breastfeeding, appropriate age to introduce home-prepared complementary food, and the risks and harmful effects of inappropriate feeding; but information on the proper use of IF is not required. It also does not have explicit safeguards to restrict idealizing text and images, and references made to proprietary products. Industry is not prohibited from producing or distributing informational materials. The 2017 Rules includes a provision that restricts health and nutrition claims, by prohibiting “messages on child health affairs, enhancement of physical and mental development of the child, improved nutritional value” of breastmilk substitutes and foods for infants and young children. There is no requirement of a warning of the risks of intrinsic contamination of powdered milk formulas.

##### 3.1.2.3. Promotion to general public

The Bangladesh BMS Act sets out to restrict all forms of advertising and promotion to the general public, including advertising, samples, free supplies, and gifts to the public, discounts at retail outlets, and direct contact with pregnant women and mothers. However, these practices are banned under the condition that they are “for the purposes of promotion or allurement of sale of any breast-milk substitutes, infant foods, complementary infant foods manufactured commercially or any accessories.” Such a caveat makes the restrictions weaker than a complete ban, in addition to the difficulties presented in proving the purpose of certain marketing practices.

##### 3.1.2.4. Health facilities and health workers

Several important prohibitions are missing from the 2017 Rules: display of CMF products in health facilities is not prohibited, and there is also no prohibition on the distribution of information or educational materials provided by manufacturers. Regarding the health system, free or low-cost supplies of CMF products and donation of equipment and services to health facilities are not explicitly prohibited. However, there is a ban on donations during emergency situations. A major limitation of the Act is the conditions placed on the prohibitions on gifts, financial incentives, and sponsorship for health workers or within the health system, and promotional activities such as contacting pregnant women and mothers. Instead of a complete ban, prohibitions are circumscribed to being applicable only when these marketing activities are “for the purposes of promotion or allurement of sale of” products in the scope. This offers inadequate protection from conflicts of interest in the health system.

##### 3.1.2.5. Labeling

Some major gaps are observed in the labeling requirements as compared to the Code. Information required for IF, such as the product should only be used on the advice of a health worker, is not addressed. Restrictions on text and images to ensure foods for infants and young children are not recommended for use before 6 months, and that they do not undermine breastfeeding, are absent. However, some parts of the labeling provisions have gone beyond the Code and required warning messages on the products including a statement “not real source of child nutrition.” The 2017 Rules have strengthened the labeling provisions, in particular the warning about intrinsic contamination and benefits of breastfeeding. The 2017 Rules also includes a provision that addresses messages relating to health and nutrition claims, which can be applied to labeling.

##### 3.1.2.6. Monitoring and enforcement

The Bangladesh Act includes a registration requirement for products covered in the scope, which serves to ensure a certain degree of labeling compliance. Sanctions and penalties are clearly outlined in both sets of legal measures. While the composition and responsibilities of the National Advisory Committee to advise on overall Code implementation are included, there is no mechanism to ensure monitoring is conducted free from conflicts of interest or to prevent members having a relationship with or interest in a CMF company.

#### 3.1.3. Bhutan

##### 3.1.3.1. Background and scope

There is no legally binding Code measure in Bhutan. The Government of Bhutan issued a policy statement in 2002 with the aim to promote, protect, and support breastfeeding. All ministries, organizations, institutes and private sectors shall support regulations in line with the South Asian Association for Regional Cooperation (SAARC) Code for the Protection of Breastfeeding and Young Child Nutrition and extend the maternity and paternity leave to facilitate the exclusive breastfeeding. Bhutan is categorized as having “no legal measures” in the 2022 Code Status Report.

The policy statement includes a section entitled “Regulation of Marketing of Food Products and Feeding Equipment Suitable for Children below Two Years of Age**,”** which contains certain provisions relevant to the Code. There are provisions that require manufacturers to seek approval from a government-approved breastfeeding committee for selling any food or feeding equipment products suitable for children below the age of two.

##### 3.1.3.2. Promotion to general public

The policy statement includes provisions that restrict promotion such as advertising in the media, as well as the distribution of free or low-cost supplies and samples to the pregnant women and mothers.

##### 3.1.3.3. Health facilities and health workers

Distribution of free or low-cost supplies, samples, and sponsorships to health workers are prohibited.

##### 3.1.3.4. Labeling

Though the provisions in the policy statement stipulate that labeling of the products must not contain anything that discourages breastfeeding nor show images other than graphics required to illustrate correct instructions on hygienic preparation, the determination on which products are applicable to these restrictions are subject to the government of Bhutan or the breastfeeding committee.

##### 3.1.3.5. Research

Disclosure of the source of funding must be included in any published research findings, and no company-funded research is allowed unless approved by the government or the breastfeeding committee.

##### 3.1.3.6. Monitoring and enforcement

The policy statement does not include provisions regarding monitoring. The policy statement is not legally-binding; hence it is not legally enforceable.

#### 3.1.4. India

##### 3.1.4.1. Background and scope

India adopted the Infant Milk Substitutes Feeding Bottles, and Infant Foods (Regulation of Production, Supply and Distribution) Act in 1992. The Infant Milk Substitutes, Feeding Bottles and Infant Foods (Regulation of production, Supply and Distribution) Rules, adopted pursuant to the Act, were passed in 1993. Both the Act and the Rules went into force in 1993. Both the Act and the Rules were amended in 2003, bringing the national law substantially aligned with the Code. The original 1992 Act and the 2003 Amendment Act are to be read together (collectively known as the IMS Act hereinafter). The IMS Act stipulates that written complaints of offences may be made by authorized officers with medical training or authorized voluntary organization (including the Breastfeeding Promotion Network of India).

The scope of India’s IMS Act is slightly narrower compared to the Code due to the upper age limit, covering CMF products up to 24 months only. It includes feeding bottles and teats but covers complementary foods (“infant foods”) from 6 months to the age of 2 years only. India is considered as being “Substantially aligned with the Code,” scoring 79 out of a possible 100 points.

##### 3.1.4.2. Informational and educational materials

The IMS Act has no explicit ban on pictures or text idealizing BMS, and no provisions to require a warning on the risk of intrinsic contamination of powdered IF. The Act states that donations of information and educational materials or equipment relating to products within the scope of the IMS Act can only be done through the health care system and must contain required details such as benefits and superiority of breastfeeding, harmful effects on breastfeeding due to partial bottle-feeding, and health hazards of improper use. However, this does not prevent companies from producing or sponsoring information and educational materials, especially on breastfeeding or infant and young child feeding in general.

##### 3.1.4.3. Promotion to general public

Apart from banning advertisement and promotion of all products under the scope of the Act to the general public, it also prohibits the supply or distribution of samples, contact with pregnant women and mothers; and offering gifts to promote products. The fact that the Act allows for the promotion of GUM allows companies to cross-promote their IF and infant and FUF of the same brand.

##### 3.1.4.4. Health facilities and health workers

The IMS Act prohibits many forms of promotion at health facilities, including financial inducements or gifts to health workers, and contributions or sponsorships for health workers and their associations for meetings, conferences, or fellowships. However, the Act only appears to prohibit the donation of “informational or educational equipment or material” but does not prohibit donations of other types of equipment or services. There is also no provision to ban health facilities hosting company events and activities; although references to specific brands of products covered in the scope are not allowed.

##### 3.1.4.5. Labeling

Regarding labeling, a major gap in the IMS Act is the absence of specific prohibition on nutrition and health claims. In other areas, the labeling provisions are stronger than the Code, especially in the text size and labeling color restrictions. There is no requirement to include a warning of risk of intrinsic contamination, although the warning text required is more detailed than that provided for in the Code in some aspects such as the outcome of improper preparation. There is no provision to prevent cross-promotion between complementary foods and CMF.

Complementary foods are defined in the scope of the IMS Act as “infant foods” being marketed as a complement to mother’s milk to meet the growing nutritional needs of the infant after 6 months and up to 2 years. Many of the provisions required in the 2016 WHO Guidance are missing, such as a statement on the importance of no complementary feeding before 6 months and the importance of continued breastfeeding for 2 years or beyond, and the prohibition of images or texts suggesting use before 6 months.

##### 3.1.4.6. Monitoring and enforcement

The IMS Act stipulates that the Ministry of Women and Child Development be responsible for the overall monitoring and enforcement, and written complaints of offenses may be made by authorized medical officers, Food Safety Officers or authorized voluntary organizations (Association for Consumer Action for Safety and Health (ACASH), Breastfeeding Promotion Network of India (BPNI), Central Social Welfare Board (CSWB), and Indian Council for Child Welfare (ICCW)). It stipulates that food inspectors can investigate suspected violations of quality standards or labeling.

It also provides sanctions, including fines and imprisonment for violations. Products may also be confiscated if they are found not compliant. However, the IMS Act does not prohibit the Government notifying a “voluntary organization” supported by a baby food manufacturer, and it does not include provisions that ensure independent monitoring is free from industry influence.

#### 3.1.5. Maldives

##### 3.1.5.1. Background and scope

Maldives adopted the Regulation on Import, Produce, and Sale of Breast-milk Substitutes to give effect to the Code in 2008. It includes a clearly outlined aim to contextualize the provisions which are substantially aligned with the Code. Not only does it cover a broad range of CMF products and complementary foods for infants and young children up to 36 months and feeding bottles and teats, but it also goes beyond the Code to cover CMF (“nutritional supplement”) for pregnant and lactating women. The Maldives is considered as being “Substantially aligned with the Code,” scoring 94 out of a possible 100 points, the highest scoring country in the region.

##### 3.1.5.2. Informational and educational materials

The Regulation stipulates that informational and educational materials regarding feeding of infants and young children, maternal nutrition, and breastfeeding (and breastmilk) are not to be donated or distributed by companies, except when they adhere to the guidelines set out in the Regulation. This incomplete ban constitutes as a weakness that can be exploited by companies. Another major weakness is the absence of a required warning about risk of intrinsic contamination of powdered IF. It however has strong provisions requiring materials that address CMF and feeding bottles to include instructions on cup-feeding and the approximate financial cost of feeding such product with the recommended quantities.

##### 3.1.5.3. Promotion to general public

The Regulation is comprehensive in prohibiting promotion to general public and clarifies that advertising activities include those on digital platforms such as “electronic transmission” and the “Internet.” Areas such as giving samples to the public and promotional devices at points of sale (retail outlets) are restricted. The target population of provisions addressing gifts and company contact through activities such as event sponsorship, contests, counselling campaigns extends beyond the Code to include the general public. However, they are written in such a way that the restrictions are only applicable when it is evident that the activities are promoting a designated product.

##### 3.1.5.4. Health facilities and health workers

Though free or low-cost supplies or samples are banned, distribution of equipment and materials in health facilities is not prohibited so long as no reference is made to any proprietary products, which is aligned with the Code but not the 2016 WHO Guidance that bans all donations of equipment and materials from companies. The Regulation does not ban health facilities to host events sponsored by companies. The Regulation outlines comprehensively the responsibilities of health workers in protecting, promoting, and supporting breastfeeding, including an overall ban for health workers to promote any designated product, and prohibition from accepting any gifts, financial or in-kind support, and fellowships from companies. These constitute a relatively strong safeguard against conflicts of interest.

##### 3.1.5.5. Labeling

The labeling provisions of the Regulation are very much aligned with the Code regarding what is required and prohibited, including the requirement of a statement on the risk of intrinsic contamination in powdered IF and prohibition of nutrition and health claims. However, there is no requirement of a statement on IF should be used only on the advice of a health worker. The provisions on restricting promotion of complementary foods prohibit such products to be marketed as suitable for below 6 months to protect exclusive breastfeeding. There are no provisions addressing cross-promotion between complementary foods and CMF.

##### 3.1.5.6. Monitoring and enforcement

The Regulation contains provisions that authorize the Maldives Food and Drug Authority (MFDA) to be responsible for overall implementation, and the Ministry of Health for monitoring and enforcement. The Regulation empowers members of the public to lodge complaints of violations. The powers and responsibilities of the National Advisory Board, which consists of various relevant government agencies, as well as nongovernmental organization representatives and members of the public, are outlined. Safeguards against conflicts of interest within the advisory board and monitoring and enforcement mechanisms are in place. Registration of products is required, which is potentially a very effective mechanism for monitoring and enforcement if carried out properly, and sanctions and penalties are clearly outlined. However, the maximum fine of around US$6,500 is not likely to deter the industry from violating the law.

#### 3.1.6. Nepal

##### 3.1.6.1. Background and scope

Nepal adopted the Breastmilk Substitutes (Marketing Control) Act 2049 in 1992, and in 1994 adopted the Breastmilk Substitutes (Marketing Control) Regulation 2051 to clarify certain provisions of the 1992 Act, including restrictions in the health system, required procedures for labeling approval, and monitoring and inspection. The scope of the Act is narrower than the Code, as it only covers CMF (BMS and other milks marketed as suitable for infants) and foods for infants and young children (“supplementary food”) up to 12 months, as opposed to up to 36 months as stipulated by the Code. Nepal is considered as being “Moderately aligned with the Code,” scoring 71 out of a possible 100 points.

##### 3.1.6.2. Informational and educational materials

The Act ensures information, such as benefits of breastfeeding, proper use of CMF marketed as BMS, and health hazards of bottle-feeding is included in the informational and educational materials. It also prohibits images or text that undermine breastfeeding. The required information on cup and spoon feeding and approximate financial cost of feeding CMF compared to cost of breastfeeding goes beyond the requirements of Code. However, there is no requirement of providing a warning on the risk of intrinsic contamination of powdered milk formulas. A major weakness is not having any provisions to completely prohibit the industry from providing information or educational materials on infant and young child feeding. Companies are allowed to provide and disseminate informational materials on infant feeding as long as there is approval from the Ministry of Health.

##### 3.1.6.3. Promotion to general public

While the provisions on promotion to the general public put a ban on advertisement of all products covered in the Act, it specifically allows companies to advertise in publications for health professionals, with conditions such as limiting the advertisements to factual and scientific matters and requiring information such as benefits of breastfeeding and risks of bottle-feeding. Company contacts with the general public are restricted. Although the restriction applies to a wider population than the Code’s “pregnant women and mothers,” such contact is only banned in health facilities.

##### 3.1.6.4. Health facilities and health workers

There is general prohibition of promotion of products covered in the Act in health facilities, including donations of items or using materials such as books or posters that refer to a proprietary product or the company name or logo. However, free or low-cost supplies are not completely banned and are allowed when requested by a health care facility and approved by the Committee.

Health workers are not allowed to promote any product covered in the Act and are obligated to report any offer of gifts or financial incentives by a company to the Committee. Although health workers are generally not allowed to accept gifts and financial or material inducements, fellowships, research grants, and sponsorship for professional meetings and attending conferences are permitted on approval by the Committee. There is no provision to explicitly restrict health facilities hosting company events and campaigns.

##### 3.1.6.5. Labeling

The Act does not have provisions to ban nutrition and health claims. It does not require warning statements on risks of intrinsic contamination of powdered IF and health hazards of inappropriate preparation. However, the Act bans images or graphics on the label or container except for illustrating methods of preparation, which provides a strong safeguard. The Act lacks labeling provisions specific to foods for infants and young children, especially those stipulated in the 2016 WHO Guidance, such as age of introduction and prohibition of any text or image that suggests use of product before 6 months. Since there are no separate provisions addressing foods for infants and young children, labeling restrictions are limited to 12 months, which is substantially narrower than the 2016 WHO Guidance which covers products up to 36 months There are no provisions addressing cross-promotion between complementary foods and CMF.

##### 3.1.6.6. Monitoring and enforcement

The Act clearly outlines the responsibilities of the Ministry of Health which chairs the multi-Ministry Committee for the Promotion and Protection of Breastfeeding, in implementation of the Act, including supervising its compliance and enforcement. The Ministry of Health also has the power, on the recommendation of the committee, to appoint inspectors to monitor compliance of companies, health facilities, and health workers. Possible sanctions for violations of the Act include fines or imprisonment, and the owners, partners of CEOs of companies are liable to these punishments. The product certification and labeling submission requirements can be effective built-in mechanisms for monitoring and enforcement. The 1994 Regulation provides further clarifications regarding monitoring health facilities and approval for product labels. There is however no safeguard to ensure monitoring and enforcement are free from commercial interest.

#### 3.1.7. Pakistan

##### 3.1.7.1. Background and scope

Pakistan adopted the Protection of Breast-feeding and Child Nutrition Ordinance in 2002. In 2009, the Protection of Breast-feeding Rules was adopted to clarify certain provisions in the Ordinance, including the constitution of the National Infant Feeding Board, required information, and prohibited content for informational and educational materials targeting health professionals, and labeling restrictions. The 2002 Ordinance and 2009 Rules are to be read together. Both were adopted as federal measures that extend to the entire country. Due to an amendment in the Constitution which decentralized the federal system, challenges in implementation ensued, and the Ordinance was devolved to the provincial level for implementation. In 2012, the Ordinance was amended at the provincial level in Punjab, which resulted in the adoption of the Punjab Protection of Breastfeeding and Child Nutrition (Amendment) Act. Several provincial regulations have been adopted since, including the Sindh Protection and Promotion of Breastfeeding and Child Nutrition Act in 2013, the Balochistan Protection and Promotion of Breastfeeding and Child Nutrition Act in 2014, and the Khyber Pakhtunkhwa Protection of Breastfeeding and Child Nutrition Act in 2015. In 2018, Punjab Province adopted the Punjab Food Authority (Baby Food) Regulations.^8^ Although the 2018 Regulations has adopted substantially more stringent regulations, such as extending the scope of breastmilk substitutes from 12 to 36 months (to include “FUF”) and include a clause to restrict cross-promotion, it is not a federal regulation and can only be implemented in the province of Punjab. However, the 2018 Regulations can lead as an example for other provinces, urging them to consider strengthening their regulatory framework.

The scope of the 2002 federal Ordinance only applies to designated products which are defined as milk or other products that replace or complement mother’s milk up to the age of 12 months, as well as feeding bottles, teats, valves for feeding bottles, pacifiers or nipple shields. Even though the provisions relevant to informational and educational materials and labeling in the 2009 Protection of Breast-feeding Rules address complementary foods, the scope is limited to only complementary foods marketed as suitable for bottle-feeding and feeding infants up to 12 months of age. Pakistan is considered as being “Moderately aligned with the Code,” scoring 73 out of a possible 100 points.

##### 3.1.7.2. Informational and educational materials

The provisions on informational and educational materials are stringent, prohibiting companies from producing or distributing any informational and educational materials relating to infant and young child feeding, except to provide scientific and information relating to designated products to health professionals which must not idealize bottle-feeding. Informational and educational materials pertaining to infant and young child feeding must be approved by the government prior to dissemination. Apart from a general ban on idealizing text or images in the 2002 Ordinance, the 2009 Rules provide clarifications on required content in materials about infant and young child feeding set out in the Code, such as the benefits and superiority of breastfeeding and the negative effect on breastfeeding of introducing partial bottle-feeding. It also requires information such as proper preparation and use of designated products and health hazards of inappropriate feeding methods to be included in materials that address feeding with designated products. Information on how to feed infants with a cup and spoon, which is not included in the Code, is required. In addition, materials addressing complementary foods must include information addressed in the 2016 Guidance, such as complementary food must only be given to infants above 6 months with a cup and spoon and not a feeding-bottle. References to any designated products or company name or logo and are not allowed in these materials. The Ordinance and the Rules are, however, missing the requirement of a warning about the risk of intrinsic contamination of powdered milk formulas.

##### 3.1.7.3. Promotion to general public

The 2002 Ordinance puts a complete ban on promotion of designated products to the general public. It has a comprehensive definition of promotion, which includes a wide range of activities, beyond advertising, that induce a person to buy or use a designated product. Apart from direct promotion by companies, the 2002 Ordinance stipulates that no person is allowed to promote or idealize any designated product by claiming that it is a substitute for mother’s milk or comparable or superior to it. This goes beyond the Code and prevents promotion by proxies such as health workers and researchers who establish affiliations with companies to further industry interests. However, the limited scope of the Ordinance means that the protection against all forms of promotion to the public is inadequate.

##### 3.1.7.4. Health facilities and health workers

The 2002 Ordinance and the 2009 Rules provide an overall prohibition of health facilities being used for promotion of designated products. There is a ban on donations or low-cost supplies to health facilities from companies, although donation of equipment and services are allowed so long as there is no reference to any designated products. Though company personnel are not allowed to contact members of the public in a healthcare facility, there are no provisions explicitly addressing the use of health facilities to host company-related events or scientific meetings sponsored by companies.

There are provisions in the 2002 Ordinance prohibiting gifts and incentives from companies to health workers and members of their family, extending to any personnel employed in a health facility, including members of the Board. This includes any material or financial incentives, such as fellowships and grants. Incentives or benefits to professional associations are prohibited, but only when they are offered for the purpose of promoting a designated product, which weakens the restriction.

##### 3.1.7.5. Labeling

The labeling provisions in the 2002 Ordinance provide general prohibitions set out by the Code, such as content that discourages breastfeeding or idealizes the product as comparable to breastmilk. However, there is no ban on nutrition and health claims. There is a complete ban on use of images and graphics, except for illustration of preparation methods. Although many of the provisions in the 2002 Ordinance and 2009 Rules on required labeling content are aligned with the Code, including a statement about the benefits and superiority of breastfeeding, specifically how mother’s milk helps prevent diarrhoea and other illnesses, there no required warning about intrinsic contamination of powdered IF.

The prohibited and required content for BMS stipulated in the Code is extended to bottle-fed complementary foods in the 2009 Rules, in addition, a statement about feeding with a cup or a spoon is safer than bottle-feeding is required. The provisions on complementary foods are strong enough to prevent such products to be marketed as suitable for below 6 months which interferes with exclusive breastfeeding. However, due to the limitation in scope of age, the provisions only apply to complementary foods up to 12 months. There is no provision addressing cross-promotion between complementary foods and CMF in the federal regulations.

##### 3.1.7.6. Monitoring and enforcement

Any person is entitled to file a complaint concerning a violation of the 2002 Ordinance or 2009 Rules to the National Infant Feeding Board which is chaired by the Ministry of Health, or a Provincial Committee. The Board can call for investigations when reports of violations are made, and the Federal Government may delegate its authority to the concerned Provincial Government where the complaint is filed. However, there is no clear monitoring mechanisms outlined in the 2002 Ordinance or 2009 Rules. In terms of sanctions, confiscation of products from manufacturers, suspension of medical license, and other penalties (after trial) are clearly stated. According to the 2002 Ordinance, the National Infant Feeding Board, which has authority over the investigation of reported violations, can include at least one member from the industry involved in manufacturing of designated products. This constitutes an inherent conflict of interest within the Board. However, it was confirmed in the Regional Workshop that steps were taken to remove member(s) of the CMF industry from the board.

#### 3.1.8. Sri Lanka

##### 3.1.8.1. Background and scope

Sri Lanka was one of the first countries to implement the Code following its adoption in 1981. The Sri Lanka Code for the Promotion, Protection and Support of Breastfeeding and Marketing of Designated Products was adopted in 1983 and amended in 2003. The scope of the Sri Lankan Code defines designated products as CMF and other milk products such as soy milk, full cream milk, and condensed milk for infants (up to 12 months); as well as bottles and teats, pacifiers, and nipple shields. The Sri Lankan Code thus permits manufacturers to promote their FUF or GUM aggressively, often with similar labeling and branding to their IFs, resulting in the cross-promotion of their IFs. Sri Lanka is considered as being “Moderately aligned with the Code,” scoring 69 out of a possible 100 points.

##### 3.1.8.2. Informational and educational materials

Companies are not allowed to produce or distribute informational and educational materials about infant and young child feeding, except when information involves the use of IF. In such case, certain information such as the benefits and superiority of breastfeeding and preparation methods is required, and text and images that idealize the product or market the product as comparable as breastmilk are prohibited. However, it does not lay down the other required content that should be included in informational materials. Critical information such as negative effect of introducing partial bottle-feeding on breastfeeding, difficulty of reversing the decision not to breastfeed, social and financial implications, health hazards of inappropriate feeding, and warning about risks of intrinsic contamination of powdered formula is not required by the Sri Lankan Code.

##### 3.1.8.3. Promotion to general public

Promotion to the general public is prohibited. Companies are not allowed to refer to designated products as equivalent, comparable, or superior to breastfeeding. Gifts, samples, and contact with pregnant women, mothers and their families are not allowed. However, the limited scope that only covers formula milk products up to the age of 12 months, the promotion of breastmilk substitutes beyond that age limit is not prohibited.

##### 3.1.8.4. Health facilities and health workers

The Sri Lankan Code stipulates that health facilities should not be used for promotion of designated products and complementary food products. Provisions pertaining to health facilities are aligned with the Code, including prohibition of mothercraft nurse services and free and low-cost supplies. Donations of equipment and other items are not completely prohibited, but references to brands or any designated products (including complementary foods) are not allowed. One major weakness is the absence of prohibition of companies using health facilities to host events, contests or campaigns. The Sri Lankan Code prohibits financial or material inducement for health workers to promote designated products and complementary foods, which is not a complete ban. Funding for research is not completely prohibited if it is approved by the Monitoring Committee (see below for more on the Monitoring Committee). There is also no prohibition on company sponsorship of professional or scientific meetings. Companies are allowed to “make contributions to a nationally recognized medical association in accordance with the objectives of Code.” These loopholes create conflicts of interest.

##### 3.1.8.5. Labeling

The labeling provisions are generally weaker than the Code. There are provisions prohibiting idealizing “terms” (e.g., maternal, humanized, etc.) and any text or image that discourages breastfeeding. However, the prohibitions only address designated products, and complementary food is not included. There is no ban on nutrition and health claims, and no provisions to ensure warning on risks of inappropriate use or preparation, and risks of intrinsic contamination, and inappropriate use before 6 months for FUF, GUM, and complementary food. There are also no requirements to include information on the recommended age for the introduction of the product, the importance of continued breastfeeding for 2 years or beyond, and the importance of not introducing complementary foods before the age of 6 months for these products.

##### 3.1.8.6. Monitoring and enforcement

The Sri Lankan Code designates a number of Ministries to be responsible for the implementation of the Code, including the Ministry of Health, Trade, Food and Marketing, Justice, and Labor. Though the Code designates the “Ministry in-Charge” to appoint a Monitoring Committee to oversee the function of implementation and monitoring, it is unclear which Ministry is primarily responsible, even though it could be assumed that the Code is under the responsibility of the Ministry of Health. There is also lack of clarity on the composition of the Monitoring Committee and the extent of jurisdiction regarding investigation and prosecution. There is no clear monitoring and enforcement mechanisms outlined in the Sri Lankan Code, therefore no requirement on such mechanisms being free from commercial interest. There are also no sanctions or penalties outlined.

### 3.2. Market value and volume trends

#### 3.2.1. Market value and volume of baby foods and CMF marketed as BMS

The overarching “baby foods” category in the market value and volume data includes CMF and foods for infants and young children such as baby wet meals, baby finger food, baby cereals, and dry meals. The CMF category is further segmented into:

1. Special formula and Starter/First Stage formula: IF (infant formula) commonly marketed for babies from birth. The figure “1” is normally used on the label. Products in this category will be referred to as IF.
2. Follow-on milk and Second Stage formula: FUF (follow-up formula) commonly marketed for babies from 6 months of age and above. The upper age indication on the product label varies country to country but is usually between 12 to 24 months. The figure “2” is normally used on the label. Products in this category will be referred to as FUF.
3. Toddler milk and Third Stage formula: GUM (growing-up milk) commonly promoted for young children between 1 to 3 years of age. The figure “3” is usually used on the label. Products in this category will be referred to as GUM.

Between 2015 and 2021, the market value of baby foods increased in Bangladesh, India, and Pakistan, while it decreased slightly in Sri Lanka. The baby foods market value is projected to increase from 2021 to 2026 in all four countries (Table 6). The market volume of baby foods increased in the four countries from 2015 to 2021 and is projected to increase to 2026 (Table 6). The market value of CMF increased from 2015 to 2021 in Bangladesh and India. The CMF market value is projected to increase from 2021 to 2026 (Table 6). The market volume of CMF increased in the four countries from 2015 to 2021 and is projected to increase to 2026 (Table 6).

**Table 6:** Value and volume of baby food and CMF by country.

	Market value (Million USD)			Market volume (Million Kg)		
	2015	2021	2026	2015	2021	2026
Baby Food						
Bangladesh	57.6	80.7	93.6	4.6	5.3	5.9
India	2,165.6	3,099.7	4,292.2	274.3	303.1	352.7
Pakistan	170.2	177.7	236.7	16.2	19.5	22.1
Sri Lanka	60.3	58.4	66.4	6.9	7.8	8.5
Asia-Pacific	24,302.1	29,507.9	39,990.5	1,246.8	1,248.8	1,376.2
Global	48,479.4	54,705.8	69,381.3	3,064.9	2,985.0	3,239.3
CMF						
Bangladesh	41.8	58.0	67.4	2.9	3.3	3.7
India	1,313.8	2,000.2	2,788.7	189.7	216.6	252.7
Pakistan	100.5	96.6	122.0	8.7	9.9	10.9
Sri Lanka	42.0	40.7	46.3	5.4	6.1	6.7
Asia-Pacific	20,851.9	25,476.0	34,856.6	979.8	984.1	1,092.3
Global	35,379.1	40,443.6	52,440.8	1,672.0	1,657.5	1,815.3

The value share of the four countries in the global baby foods sector increased from 5.1% in 2015 to 6.2% in 2021 and is expected to increase to 6.8% in 2026 (Figure 2). Similarly, the volume share of the four countries in the Asia-Pacific region increased from 10.1% in 2015 to 11.6% in 2021 and is expected to increase to 11.7% in 2026 (Figure 2). The volume share of the four countries in the global baby foods sector increased from 9.9% in 2015 to 11.2% in 2021 and is expected to increase to 12.0% in 2026. Similarly, the share at the regional level is expected to increase from 24.2% in 2015 to 26.9% in 2021 and is expected to reach 28.3% in 2026.

**Figure 1:**
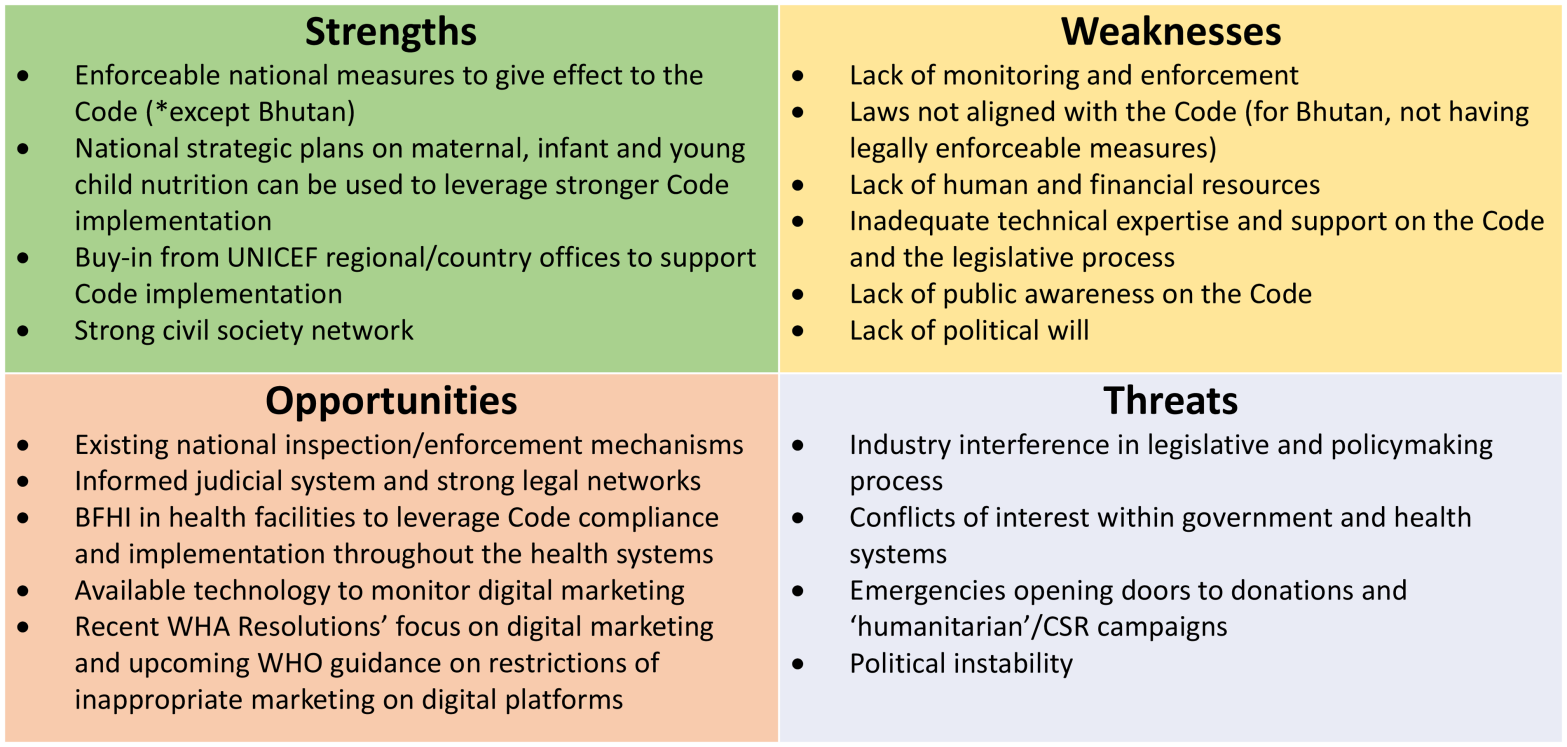
Summary of SWOT analysis.

**Figure 2:**
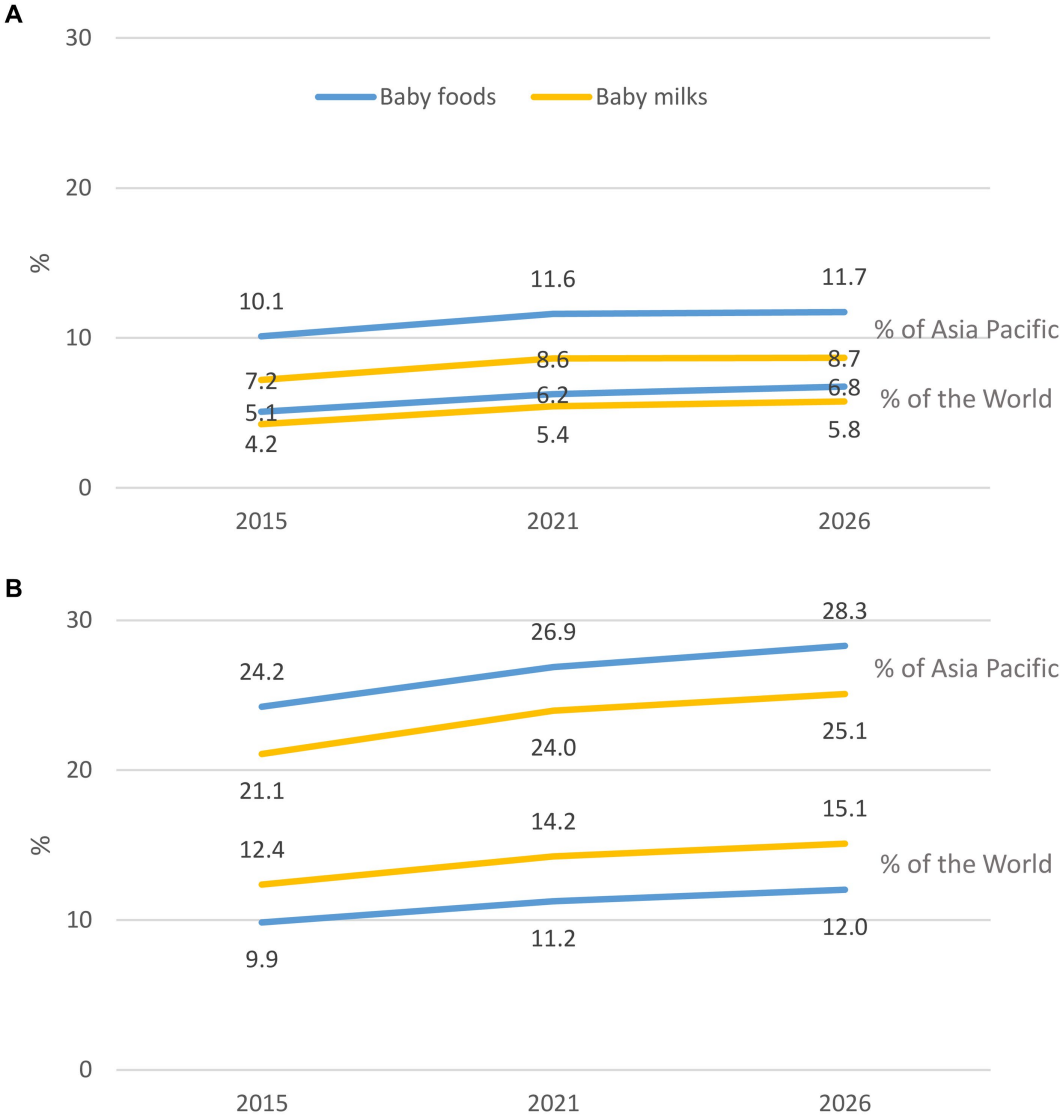
Percentage of the contribution of value **(A)** and volume **(B)** of the four countries to Asia Pacific region and the world.

The value share of the four countries in the global CMF sector increased from 4.2% in 2015 to 5.4% in 2021 and is expected to increase to 5.8% in 2026. Similarly, the share of CMF at the regional level increased from 7.2% in 2015 to 8.6% in 2021 and is expected to increase to 8.7% in 2026. The volume share of the four countries in the global CMF sector increased from 12.4% in 2015 to 14.2% in 2021 and is expected to increase to 15.1% in 2026. Similarly, the share at the regional level increased from 21.1% in 2015 to 24.0% in 2021 and is expected to increase to 25.1% in 2026.

#### 3.2.2. Value and volume trends of baby foods and CMF segmentation by country

**Figure 3:**
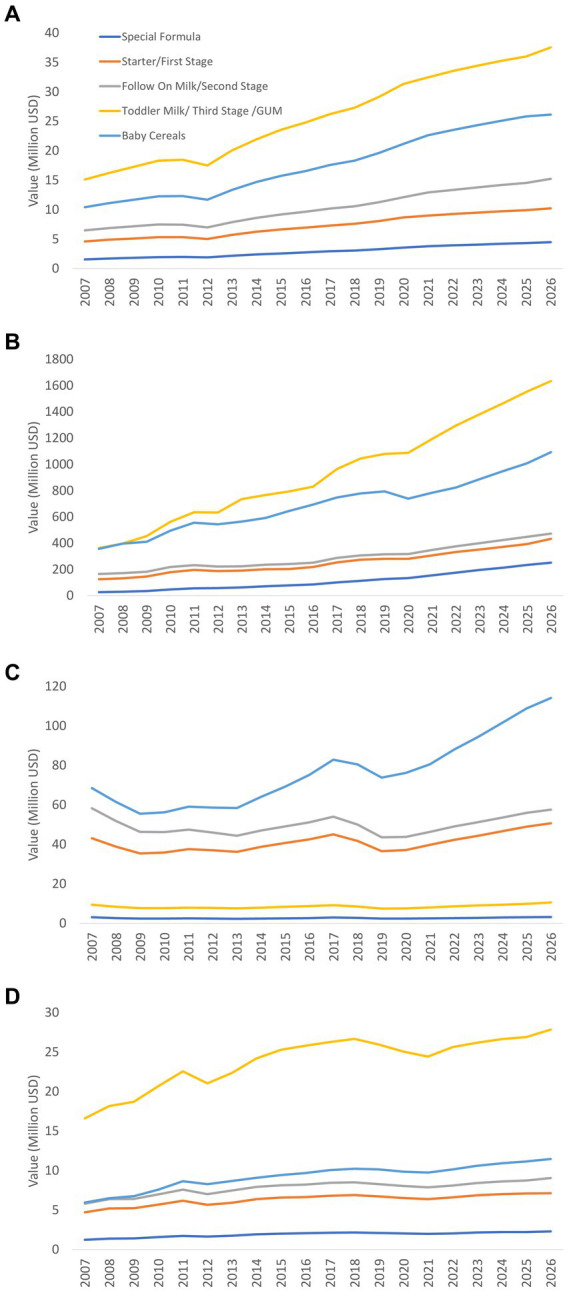
Value trends of baby foods and CMF segmentation in Bangladesh **(A)**, India **(B)**, Pakistan **(C)**, and Sri Lanka **(D)** (actual 2007–2021, projection 2022–2026).

There were overall increased trends in all segmentations of CMF, especially GUM in Bangladesh, India, and Sri Lanka (Figure 3). Sales of foods for infants and young children, in particular baby cereals, also increased at a high rate in Bangladesh and India (Figure 3). However, the growth rate for select segmentations of CMF decreased in the late 2000s (Bangladesh, Pakistan, and Sri Lanka) and 2010s (India, Pakistan, and Sri Lanka) (Figure 3).

Overall, GUM, followed by baby cereals, accounted for a large portion of baby foods in Bangladesh, India, and Sri Lanka (Figure 3). In contrast, in Pakistan, IF and FUF, rather than GUM, were the major baby CMF (Figure 3).

**Figure 4:**
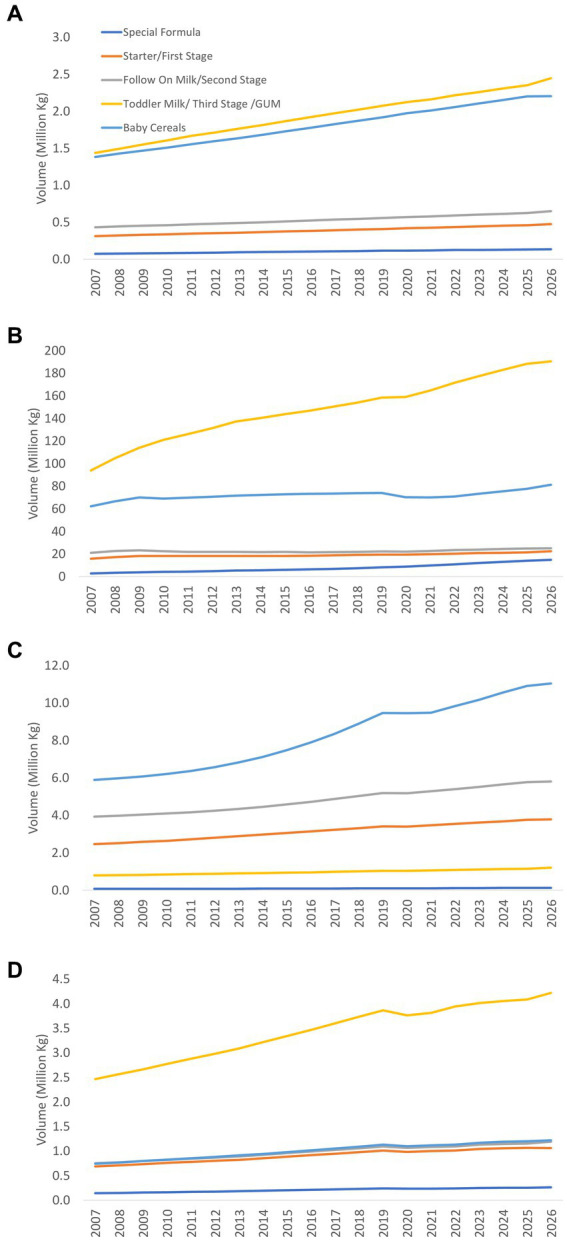
Volume trends of baby foods and CMF segmentation in Bangladesh **(A)**, India **(B)**, Pakistan **(C)**, and Sri Lanka **(D)** (actual 2007–2021, projection 2022–2026).

Very similar trends were found in the volume of CMF. However, the rate of increment is quite steady and did not decrease in the late 2000s and 2010s, with the exception of GUM in Sri Lanka (Figure 4).

#### 3.2.3. *Per capita* expenditure and consumption of CMF in India

**Figure 5:**
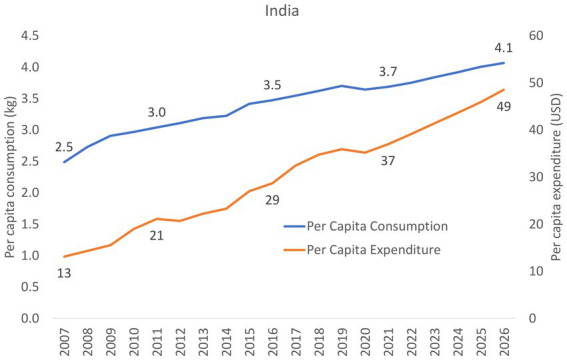
Trends of *per capita* expenditure and consumption of CMF in India (2007–2026).

*Per capita* expenditure on CMF in India grew from $13 in 2007, $29 in 2016 to $37 in 2021 (Figure 5), which was lower than the global average ($167) and the regional average ($152). *Per capita* expenditure CMF in India is projected to increase further to reach $49 in 2026 (Figure 5). Data from the other three countries were not available.

*Per capita* consumption of CMF in India increased slightly from 2.5 kg in 2007, 3.5 kg in 2016 to 3.7 kg in 2021 (Figure 5), which was lower than the global average (9.3 kg) and the regional average (6.7 kg). *Per capita* consumption of CMF in India is anticipated to increase further to reach 4.1 kg in 2026 (Figure 5). Data from the other three countries were not available.

#### 3.2.4. Distribution channels of baby foods and CMF

**Figure 6:**
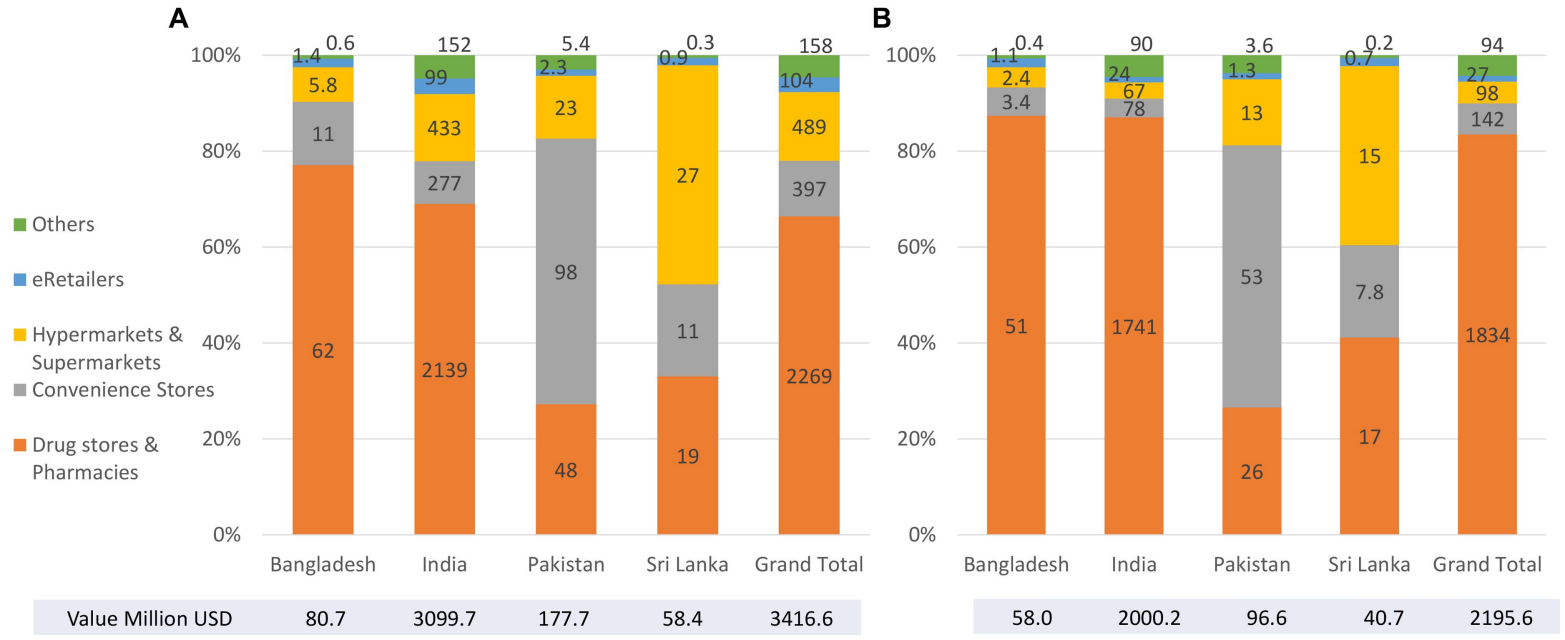
Key distribution channels of baby foods **(A)** and CMF **(B)** by country (2021).

In 2021, key distribution channels of baby foods were drug stores and pharmacies in Bangladesh and India, convenient stores in Pakistan, and drug store, pharmacies, hypermarket, or supermarket in Sri Lanka (Figure 6). A similar pattern was found in distribution channels for CMF.

### 3.3. Policy implementation and sales volumes and values

The amendment of the national measures in Bangladesh (in 2017) and the Punjab Food Authority (Baby Food) Regulations adopted in Pakistan (in 2018) occurred between the years with available sales value and volume data (from 2007 to 2021). In Bangladesh, there has not been any obvious change in sales and volume of CMF and baby cereals after the adoption of the 2017 Rules.

In Pakistan, the drop in the sale of first and second stage CMF (IF and FUF) and baby cereals started in 2017 and continued through to the adoption of the new Regulations in 2018, until 2019 when there was an increase for first and second stage CMF (IF and FUF) and baby cereals. The sales volume of baby cereal in Pakistan leveled off from 2019 (a year after the implementation of the adoption of the Regulations in 2018).

## 4. Discussion

The findings above provided an analysis of gaps and strengths of the Code-based national measures of Afghanistan, Bangladesh, Bhutan, India, Maldives, Nepal, Pakistan, and Sri Lanka; and sales volumes and values of CMF and complementary food products of Bangladesh, India, Pakistan, and Sri Lanka.

### 4.1. Overview of code and national laws

#### 4.1.1. Scope

The Code (including the 2016 WHO Guidance) applies to marketing of all CMF products that function as BMS (up to 36 months of age), any other products marketed for feeding infants up to 6 months, feeding bottles and teats, and all commercial foods and non-formula milk beverages marketed for infants and children 6 months to 3 years of age.

The Maldives’ Regulation has met the scope set out in the Code, and even goes beyond it to cover CMF for pregnant and lactating women. The scope of India’s IMS Act only covers CMF products up to 24 months. National laws of Nepal, Pakistan, and Sri Lanka all only cover CMF products up to 12 months, leaving GUMs uncovered. Although the 2022 Code Status report indicates that Bangladesh’s legal measures cover CMF up to 60 months, a discrepancy exists, and the lack of clarity in the age range of CMF covered in the scope could render an interpretation of a six-month upper age limit that is favored by industry. When national regulations only cover CMF products up to the age of 12 or 24 months, that leaves companies free to promote their FUF or GUM. Often the branding and labeling are deliberately similar to that of their IFs. This results in the cross-promotion of their IFs, which undermines both exclusive and continued breastfeeding (30).

#### 4.1.2. Information and education materials

Governments have ultimate responsibility to ensure information provided on infant and young child feeding is objective and consistent. There are key points that must be included in information and education materials, including benefits and superiority of breastfeeding and negative effect on breastfeeding of introducing partial bottle-feeding. Specifically for IF, statements such as health hazards of inappropriate use and warning on risk of intrinsic contamination (for powdered formula) are required. There are also prohibited contents such as reference to proprietary products and idealizing images or text. The 2016 WHO Guidance gives countries extra leverage to introduce stronger provisions, prohibiting dissemination of informational or educational materials by companies (14). In the region, Afghanistan’s national law is most aligned with the Code in this area, except the requirement on warning on risk of intrinsic contamination of powdered formula is absent.

#### 4.1.3. Promotion to public

The term promotion was not clearly defined in the 1981 Code. The 2016 WHO Guidance defines “promotion” broadly to include messages designed to encourage the purchase or consumption of a product or raise awareness of a brand. It also clarifies that a reference to a brand name is not necessary for it to be considered as promotion.

There are loopholes in the Code that prevent protection of all potential targets of promotion. The Code only bans advertising directed “to the general public,” which could be misused to indirectly suggest that groups such as health professionals are excluded. Some provisions only apply to “pregnant women, mothers or members of their families.” There is also ambiguous language such as a ban on gifts “which may promote the use of BMS or bottle feeding, allowing gifts that do not appear overtly promotional. Though the Code prohibits company contact with pregnant women and mothers, it is limited to only “marketing personnel” (14).

Countries such as India and Bangladesh have improved on the ambiguity by prohibiting all forms of advertising and promotion without limitation. National laws of Afghanistan, Bangladesh, India, Maldives, Nepal, Pakistan, and Sri Lanka have included provisions on advertising, providing samples to the public, promotional devices at points of sale (retail outlets), and company contact with mothers. The policy statement issued by the government of Bhutan does not mention promotion at retail outlets but requires approval from the government (or its appointed breastfeeding committee) for sale of covered products.

Even with legal measures that cover a wide range of promotions, they are only applicable as far as the coverage of the scope. For instance, the Sri Lankan Code and Pakistan’s national measures do not cover GUMs. Evidence shows that apart from the public being exposed to the promotion of formula products through social media, the promotion of GUMs, which have similar branding to the company’s IF, also results in the cross-promotion of the entire range of the company’s CMF, undermining breastfeeding (33). In Pakistan, during the COVID-19 pandemic, companies used GUMs to reach out to mothers via social media, using immunity claims and fear-provoking questions to suggest their CMF products can protect children from COVID.

#### 4.1.4. Health systems

Due to the ‘endorsement by association’ effect conferred on companies; health facilities are fertile ground for promotion. Our findings show that health facilities and pharmacies are where sales are highest.

While the national laws of the seven countries have included general prohibitions on using health facilities for promotion, each has slightly different coverage. The seven countries with legal measures have provisions that restrict display of placards or posters concerning covered products. Only India and Bangladesh have provisions addressing the display of products in the scope of the national law. The laws of Bangladesh, Maldives, and Pakistan do not specify a complete ban on distribution of materials provided by companies in health facilities, prohibiting only materials that reference a designated product. The use of health facilities to host company events is addressed in the 2016 WHO Guidance (34), but it is not specifically addressed in the laws of India, Maldives, Nepal, Pakistan and Sri Lanka.

WHA Resolution 47.5 [1994] prohibits donations or low-cost supplies of CMF and other products that are covered in the scope of the Code in the health systems. The seven countries with legal measures all restrict manufacturers offering low-cost or free supplies (donations) of CMF and other related products covered in their national laws within the health systems. However, the degree of restrictions vary, for instance, restrictions in Nepal are not complete bans as the 1992 Act allows donations so far as they are approved by the Committee for the Promotion and Protection of Breastfeeding.

How the health system and health workers foster or discourage breastfeeding is one of the key determinants for successful initiation and maintenance of breastfeeding. Companies are eager to offer sponsorship for conferences and research, and forge strong financial links with medical establishments, professional associations, and public health agencies. Currently, 38 percent of national paediatric associations around the world receive funding for their conferences from CMF manufacturers (35). India has adopted provisions to restrict company sponsorship for health workers’ meetings, conferences, or fellowships. The seven countries with legal measures have all included provisions that restrict financial, or material inducement aimed at promoting products covered in the scope, with varying degrees of restrictions. Sponsorships such as fellowships, study tours, research grants, attendance at professional conferences are also not allowed, except for Nepal, which requires disclosure rather than a ban. While prohibiting distribution of samples, none of the countries in this study enacted a complete ban on donations of equipment or services to health facilities; for example, Bangladesh, India, Nepal, and Sri Lanka prohibit such donations only when a proprietary product is mentioned.

#### 4.1.5. Emergencies

The region of South Asia is especially vulnerable to natural disasters and other emergencies (36). Companies capitalize on emergencies by providing donations of CMF and related products to widen markets and foster public image (22, 37, 38). Donations, and the indiscriminate distribution, are extremely harmful in emergency contexts, often contributing to child morbidity and mortality (37, 39). It is difficult to ensure proper usage, sufficient supplies, and access to resources for safer bottle-feeding (e.g., clean water) in emergencies, Hence the uncontrolled distribution of products is harmful even to infants and children for whom breastfeeding is impossible (37). WHA Resolution 63.23 [2010] and the Operational Guidance on Infant and Young Child Feeding in Emergencies (OG-IFE) (40) stipulate that donations of CMF, complementary foods and feeding equipment should not be sought or accepted, and supplies should be procured through official channels and distributed based on strict criteria. Out of the seven countries with legal measures, only Bangladesh and Sri Lanka have provisions addressing restrictions on donations during emergencies. Sri Lanka’s Code outlines strict criteria for donations instead of a complete ban.

#### 4.1.6. Labeling

Labeling provides necessary information about the product’s content and proper use. Companies also use it to promote their products through idealizing text or images, or omission of warning. For information that is required to be on labels of IF, the countries with legal measures have provisions to ensure a statement on superiority of breastfeeding and instructions for appropriate preparation. Laws of Nepal, Pakistan, and Sri Lanka do not specify the requirement of a warning on the health hazards associated with inappropriate preparation. All seven countries with legal measures ban idealizing images on labels, Nepal and Pakistan have even gone beyond the Code, putting a complete ban on use of images and graphics, except for illustration of preparation methods.

Powdered formula can become contaminated during the manufacturing process with dangerous bacteria. WHA Resolution 58.32 [2005] requires governments to ensure labels include warnings on the risks of intrinsic contamination of powdered IFs, so the public is fully informed (14). Only Bangladesh and Maldives have adopted provisions to include such warning. Currently there are product liability lawsuits in the United States that implicate possible associations between IF use and premature babies being diagnosed with necrotizing enterocolitis (NEC) that led to death or required surgical intervention (30). These lawsuits shed light on the importance of manufacturers providing appropriate warnings on their products so parents can make informed decisions.

Health claims, which are mostly unsubstantiated (41), have been used as promotional and premiumization tools, misleadingly conveying physiological, developmental, and cognitive benefits (Becker et al. (42)). Claims on immunological protection has been especially rampant since the COVID-19 pandemic (22). Nutrition and health claims are prohibited by WHA Resolutions 58.32 [2008] and 63.23 [2010] except where specifically provided for in relevant Codex Alimentarius standards or national legislation (14). Out of the countries with legal measures, only Afghanistan, Bangladesh, and Maldives have provisions that restrict health claims. However, due to the gaps in the scope of Afghanistan’s and Bangladesh’s legal measures, it means that health claims can still appear on products that are not covered in the scope.

#### 4.1.7. Foods for infants and young children

Regarding foods for infants and young children (complementary foods), Afghanistan and Maldives have provisions that ensure information such as recommended age for introducing the product, no complementary feeding before 6 months, and the importance of continued breastfeeding for 2 years or beyond is provided on the labels. They also have provisions that prohibit image or texts that suggest use before 6 months, messages that discourage breastfeeding, and professional endorsement. Nepal and Pakistan have restrictions on images or texts that suggest use before 6 months and messages that undermine breastfeeding. Bangladesh restricts promotion of “commercially manufactured complementary foods” (foods for infants and young children) up to 5 years, going beyond the 2016 Guidance’s scope. Sri Lanka is the only country that has no provisions that specifically place prohibitions on complementary foods labeling.

#### 4.1.8. Conflict of interest

Conflicts of interest within the health system and government, such as industry sponsorship of health programs and industry’s participation in policymaking and monitoring, can impede the adoption of strong laws and proper enforcement. This study reveals two main problems. Firstly, for some countries, their law specifies that financial or material inducements are not allowed only if they are used for promoting products covered in the law. This creates situations where financial or materials inducements could arguably be allowed so long as they are not explicitly specified with a purpose to promote designated products. Secondly, the definition of conflicts of interest may not be clear, allowing room for companies to establish ambiguous ‘partnerships’ or ‘corporate social responsibility’ campaigns that are fraught with conflicts of interest. The 2016 WHO Guidance reinforces with clearer conflicts of interest safeguards within the health systems.

#### 4.1.9. Monitoring and enforcement

Code monitoring is vital to identifying violations and effective enforcement. It provides basis for reviewing and adopting new laws. Except for Bhutan and Sri Lanka, all other countries have identified a specific agency responsible for monitoring and enforcement. The Code stipulates that monitoring is the responsibility of national governments, and WHA Resolution 49.15 highlights that it should be independent and free from commercial influence (14). Only Afghanistan and Maldives have included provisions to ensure monitoring is independent and free from industry influence; such provision is absent from the laws in other countries. According to the findings from the Regional Workshop, all countries in the region have been lagging in monitoring. Only India reported there is some ongoing monitoring, and another country, Sri Lanka, has had “piece-meal” monitoring over the years (28).

All countries in the region reported a lag in enforcement. Most of the countries reported there is a lack of clarity on role and coordination with government agencies, lack of allocated resources, as well as an absence of an effective mechanism (30). Recent studies on Bangladesh, India, and Maldives also corroborated with the reported lack of enforcement (20, 21, 23).

No legal measures are in place in Bhutan to give effect to the Code through enforcement. Provisions included in the 2002 Policy Statement are insufficient to restrict inappropriate marketing, even if they were legally binding (30).

### 4.2. Digital marketing

Many countries in the Regional Workshop expressed uncertainty regarding regulation of digital marketing - even though much of it is explicit promotion to the public. Legal measures, especially those drafted and adopted in earlier years, often do not explicitly ‘single-out’ digital marketing as a separate category, but it is often implicit in provisions addressing advertising and promotion to the public. WHA Resolution 69.9 [2016] addresses the pervasive promotion that has rapidly increased over social media in recent years (14), urging all media to comply with the Code.

### 4.3. The sales and volume of baby foods and CMF

Overall, there is an increasing trend in the sales and volume of baby foods and baby milk in South Asia. The trend was also found in the world and other regions, especially in Asia (24, 43). As shown in Figures 3, 4, the sales value and volume of the IF category (which includes special formula and starter/first stage formula) in Bangladesh, India, and Sri Lanka have been consistently lower than the other CMF products marketed for older infants and young children (e.g., FUF and GUM), and GUM has accounted for a large portion of CMF sales in these countries. IF is the only category that is commonly covered by the scope of the national measures of these three countries, while GUM is not sufficiently covered and may provide loopholes for promotion. However, statistical inference to indicate Code implementation’s impact on CMF sales cannot be drawn based on existing data and current methodology, which is a limitation of this study.

Countries with a larger number of annual live births (i.e., India: 24,068,000, Pakistan: 6,046,000, Bangladesh: 2,890,000, Sri Lanka: 326,000) (44) have higher absolute sales and volumes of baby foods and CMF as anticipated. There is a marked increase in the *per capita* consumption of CMF in India in volume from 2.5 kg in 2007 to 3.7 kg in 2021 (corresponding to USD 13–37). Although the volume is lower than in Asia (11.7 kg–29.3 kg per infant 2005–2017) and the world (16.0 kg–28.5 kg per infant 2005–2017) (43), India is the second largest and will soon become the largest country in the world (44).

The relative contribution of the volume of baby foods and CMF sold in South Asia is about 2–3 times as high as that from value compared to the Asia Pacific region and the world. The findings suggest that lower-price baby foods and milks are sold in South Asia. A potential explanation could be local production of and lower standards on baby foods and CMF mandated. In China, increased standards on CMF shift the sale and consumption to specific brands and increased the price (45). Some fluctuations in total value in select years have been observed, but a steady increase in trend in volume. The company might reduce the interest margin to reduce its price to keep the sale volume stable and increase consumption.

### 4.4. Analysis of the 2022 Code status report

The 2022 Code Status Report provides a clearly categorized format that allows for quick reference and comparison among countries. The use of a scoring algorithm ‘checks off’ itemized content in the text of the legal measures. However, it does not adequately reflect the quality and level of restrictions offered as they are often couched in complicated legalese. It is difficult for the scoring algorithm to take into account every specificity of the provisions of the Code and relevant resolutions when analysing national Code legislations (30). Hence, provisions such as prohibition of cross-promotion between CMF and complementary foods (2016 WHO Guidance) and the ban on donations of products in the scope of the Code in emergencies as stipulated in Resolution WHA 63.23, are not included in the scoring criterion. For monitoring and enforcement, the algorithm only considers selected aspects of monitoring - whether the national legislation identifies who is responsible for monitoring compliance, defines sanctions for violations, and requires that monitoring and enforcement should be independent, transparent, and free from commercial influence. These three criteria do not sufficiently indicate an effective monitoring or enforcement system as it does not provide information as to whether monitoring and enforcement mechanisms are implemented or integrated into existing systems (30). For a more comprehensive and in-depth analysis, it is important to gather information on implementation ‘on the ground’. The legal measures also need to be examined qualitatively. Hence the systematic content analysis outlined by Salehijam (27), a research method that is replicable and commonly applied by social scientists in the analysis of a variety of texts, was used in this study.

### 4.5. Limitations

The challenges in assessing national measures, as well as the lack of monitoring and enforcement on the ground, create additional difficulties in making direct associations between national regulations and sales. The study by Piwoz and Huffman (26) acknowledges that factors other than Code implementation, such as women’s labor force participation rate, likely also contribute to impact CMF sales. This study acknowledges that factors such as public health policy, access to breastfeeding support, maternity protection, and social beliefs, may also influence breastfeeding practices, and thus the sales volume of CMF. Existing literature is not able to provide accurate data on spending on CMF marketing (46), but it is speculated that spending on marketing is on the rise, and significantly exceeding government budgets on breastfeeding promotion (47, 48). Future research can explore a more direct relationship that is between the prevalence of aggressive marketing and Code implementation status.

## 5. Recommended actions

The following recommended actions are based on findings from reviewing national measures, sales data analysis, and feedback during the Regional Workshop.^9^

1. Develop plans appropriate to the national context to close the gaps between national measures and the Code^10^:
  ■ Bring the age range of the scope of CMF and foods for infants and young children in national regulations on par with the 2016 WHO Guidance as a minimum (36 months, including IF, FUF, and GUM).
  ■ Restrict health and nutrition claims and endorsements for products in the scope of the Code. Also, care should be taken with products out of the scope of the Code, such as CMF for pregnant and lactating women.
  ■ Implement WHA Resolution 58.32 [2005] in national measures to ensure warning on risks of intrinsic contamination of powdered formula is conveyed through labeling and information materials.
  ■ Ensure conflicts of interest in the health system are addressed in national measures, in in line with relevant WHA resolutions and the 2016 WHO Guidance. Implement enforceable code of conduct to ensure activities from health professionals are free from industry influence.
  ■ Ban donations in emergencies by ensuring Resolution WHA 63.23 [2010], which endorses the OG-IFE, is incorporated into national measures and emergency management policy.
  ■ Develop compliance and management policy must be developed and implemented to avoid conflicts of interest within governments in their policymaking and law implementation processes.
  ■ Ensure legal measures provide authority for all necessary agencies to implement, monitor and enforce all aspects of the law. The designated government agencies must monitor compliance and identify violations, and corrective actions must be taken through clearly specified administrative or legal sanctions.
  ■ Marketing practices are mostly planned centrally. Monitoring and enforcement can be conducted in a targeted manner, integrated into existing inspection systems. Labeling and marketing on digital platforms are manageable starting points as they are more visible for detection.
2. Effort is needed to strengthen technical and financial support in monitoring and enforcement, a barrier identified by the countries.
3. Conduct monitoring on digital platforms to identify enforcement efforts and whether existing measures can be used to tackle violations (49). The use of novel technology such as artificial intelligence should be integrated. As demonstrated through the Virtual Violations Detector (VIVID) used in Vietnam (https://code.corporateaccountabilitytool.org/vietnam), such technology is able to aid monitoring and enforcement.

5.3. Sensitize the legal and jurist networks to the importance of strengthening Code implementation and cultivate support. High level political support at the national level also needs to be galvanized.

## 6. Conclusion

The assessment of Code implementation and sales of breastmilk substitutes in South Asia highlights the existing gaps in implementation despite the presence of legal measures. The narrow scope of CMF in national laws, inadequate conflicts of interest safeguards, and the lack of effective monitoring and enforcement mechanisms are some of the factors that potentially contribute to the persistent aggressive marketing practices that hinder breastfeeding. ‘Good’ Code implementation alone is not enough – but it is a critical first step to help build an enabling environment for breastfeeding to stand a fair chance. The region is faced with compounded challenges in tackling malnutrition and mortality, as well as difficulties in implementing and enforcing regulatory measures. Yet, it is also filled with resources and systems to overcome these difficulties. While all sectors working to protect breastfeeding and maternal and child health should have the political courage to call out and withstand pressure from industry - governments are the ultimate duty-bearers to hold companies accountable through adopting and enforcing laws, and ensuring policy- and law-making is free from commercial influence.

## Data availability statement

The original contributions presented in the study are included in the article/Supplementary material, any further inquiries can be directed to the corresponding author.

## Author contributions

CC, VS, TN, and KS were involved in the conceptualization, methodology and analysis. VS, ZM, and CC obtained the relevant data. CC, VS, and TN led the drafting. KS provided the legal input. KK, PZ, and KS provided the thorough review. KK, PZ, and TF provided the project administration. VS, ZM, and TF were involved in the funding acquisition. All authors have provided the technical input, read, and agreed to the published version of the manuscript.

## Footnotes

^1^More recently the term commercial milk formula (CMF) is being used to describe all milk products marketed for use by children up to the age of 36 months (3). The term CMF will be used hereinafter except for contexts relevant to national legal measures where BMS is used specifically.

^2^Although the study period was between June to December 2022 (up until the first submission), the review process has allowed revisions and updates to be made until August 2023.

^3^Bhutan does not have any legal measures to give effect to the Code.

^4^The workshop was conducted with the aim to (i) identify gaps between national measures and the Code, (ii) identify major strengths and weaknesses of their national measures, and discrepancies in the scoring of the 2022 Code Status Report, (iii) perform a Strengths, Weaknesses, Opportunities, and Threats (SWOT) Analysis (29) of the national regulatory framework, and (iv) to develop a national Code action plan based on the SWOT analysis.

^5^The analysis informs countries of key areas for action planning according to the national context. While each country’s situation is unique, common themes have emerged and were summarized in Figure 1.

^6^ www.globaldata.com

^7^More details on CMF segmentation will be found in the Results section.

^8^Even though the period of this study is June to December 2022, in August 2023, the Sindh assembly passed the Protection and Promotion of Breast Feeding and Young Child Nutrition Act, after the second regional Code workshop which took place in May 2023. The Sindh Act is included here as additional information because this manuscript was still being reviewed and revised during the time of its adoption.

^9^Detailed information that outlines revisions that can be made to current regulatory frameworks in the seven countries with legal measures is listed in Supplementary Table S1.

^10^For Bhutan, enforceable legal measures need to be adopted to give effect to the Code.
