# Supplementary material for: Law matters – assessment of country-level code implementation and sales of breastmilk substitutes in South Asia
Source: Front Public Health. 2023 Oct 23;11:1176478. doi: 10.3389/fpubh.2023.1176478 (PMC10626485; doi:10.3389/fpubh.2023.1176478)
Supplement: Supplementary file 1 [file Table_1.DOCX]

**Supplementary material 1**

The table below provides an overview of the revisions that can be made to current regulatory frameworks in the seven countries in South Asia with legal measures to give effect to the Code. Bhutan has not been included in this table as it does not have any legal framework in place.

|  | **AFGHANISTAN** | **BANGLADESH** | **INDIA** | **MALDIVES** | **NEPAL** | **PAKISTAN** | **SRI LANKA** |
| --- | --- | --- | --- | --- | --- | --- | --- |
| **Scope** | Cover all BMS up to 36 months | Cover all BMS up to 36 months | Cover all BMS up to 36 months | Complimentary foods and feeding bottles and teats. | Cover all BMS up to 36 months | Cover all BMS up to 36 months | Cover all BMS up to 36 months |
| **Monitoring & enforcement** | Establish Government monitoring system which satisfies NetCode requirements. | Establish Government monitoring system which satisfies NetCode requirements. | Establish Government monitoring system which satisfies NetCode requirements. | Examine M&E system to ensure satisfies NetCode requirements | Examine M&E system to ensure satisfies NetCode requirement | Establish Government monitoring system which satisfies NetCode requirements. | Establish Government monitoring system which satisfies NetCode requirement and specify sanctions. |
| **Info. materials** | Include warning on intrinsic contamination (IC) | Prohibit industry materials and include info on proper use of IF when necessary and warning on IC | Prohibit industry materials and include warning on IC | Prohibit industry materials and include warning on IC | Prohibit industry materials and include warning on IC | Info. Requirements in the Rules need to extend to ALL info. materials and need to include warning on IC. | Include requirements of Article 4.2 of the International Code and warning on IC. |
| **Promotion to public** | Prohibit direct or indirect contact with mothers | Despite prohibitions, public still exposed to promotion. Requires effective monitoring and enforcement | Prohibit promotion of “growing-up milks” to stop companies cross-promoting their IF and FUF of the same brand. | Examine the true extent to which the public is protected from digital and on-line marketing | Prohibit industry contact with mothers anywhere; not just in the health care system | Prohibit promotion of “growing-up milks” to stop companies cross-promoting their IF and FUF of the same brand. | Prohibit promotion of “growing-up milks” to stop companies cross-promoting their IF and FUF of the same brand. |
| **Promotion in health system** | Prohibit company-paid employees in health system | Prohibit display of BMS | Prohibit company-paid employees in health system | Prohibit use of the health facility by companies to host events, contests or campaigns. | Prohibit company-paid employees in health system and use of the health facility by companies to host events, contests or campaigns. | Prohibit industry materials, company-paid employees in health system and use of the health facility by companies to host events, contests or campaigns. | Prohibit use of the health facility by companies to host events, contests or campaigns. |
| **Engagement with health system** | Prohibit company donations of equip. or services | Prohibit company donations of equip. or services, free supplies of BMS, sponsorship of meetings. | Prohibit company donations of equip. or services. | Prohibit company donations of equip. or services. | Prohibit company donations of equip. or services, free supplies of BMS, and scholarships and research grants. | Prohibit company donations of equip. or services, free supplies of BMS, sponsorship of meetings. | Prohibit company donations of equip. or services, free supplies of BMS, sponsorship of meetings, and contribution to medical associations. |
| **Labelling** | Prohibit health claims on all BMS and include warning of intrinsic contamination | IF labels to state only for use on advice of health worker. FUF labels to recommend age of use and no images or text undermining breastfeeding | Prohibit health claims on all BMS and include warning of IC. FUF labels to recommend age of use and no images or text undermining breastfeeding | IF labels to state only for use on advice of health worker. Warning on the risk of IC should be in contrasting colour of a size not less than 1/3 the size of the characters in the product name. | Prohibit health claims on all BMS and include warning of IC. FUF labels to recommend age of use and no images or text undermining breastfeeding | Prohibit health claims on all BMS and include warning of IC. FUF labels to recommend age of use and no images or text undermining breastfeeding | Prohibit health claims on all BMS and include warning of intrinsic contamination. FUF labels to recommend age of use and no images or text undermining breastfeeding |
